# Cation modification of montmorillonite regulates the retention of biomass-derived artificial humic substances

**DOI:** 10.1039/d6ra09407d

**Published:** 2026-11-04

**Authors:** Khatereh Pakzad, Giulia Ischia, Markus Antonietti, Jiyong Kim, Vera Susanne Rotter, Alexander Volikov

**Affiliations:** a Department of Colloid Chemistry, Max Planck Institute of Colloids and Interfaces Am Mühlenberg 1 14476 Potsdam Germany Khatereh.Pakzad@mpikg.mpg.de; b Functional Materials and xDevices, Fraunhofer Institute for Applied Polymer Research Potsdam (IAP) 14476 Potsdam Germany; c Department of Environmental Technology, Chair of Circular Economy and Recycling Technology, Technische Universität Berlin 10623 Berlin Germany

## Abstract

Interactions between biomass-derived organic matter and clay minerals are important for understanding organo-mineral associations and evaluating strategies for organic carbon retention. In this work, artificial humic substances (AHS) were prepared from pear pomace and pine bark as biowastes through hydrothermal humification. The resulting AHS were then contacted with pristine and cation-modified montmorillonite (MON) to investigate the effect of Na^+^, Ca^2+^, and Fe^3+^ modification on AHS adsorption. AHS adsorption behavior was evaluated over a concentration range of 0.5–25 g L^−1^, and the resulting materials were characterized using complementary structural, textural, and surface-sensitive techniques. Carbon retention increased in the order Na-MON < Ca-MON < Fe-MON, with values of 316, 413, and 546 mg C per g clay for Na-MON, Ca-MON, and Fe-MON, respectively. Among the models evaluated, the Sips model exhibited the best agreement with the experimental adsorption isotherm data, suggesting heterogeneous adsorption behavior and a finite retention capacity within the investigated concentration range. The pseudo-second-order model better described adsorption kinetics than the pseudo-first-order model. Across four adsorption–desorption cycles, Fe-MON exhibited the lowest desorption capacity, decreasing from 58.37 mg C g^−1^ in the first cycle to 12.83 mg C g^−1^ in the fourth, while Na-MON showed the highest relative desorption, decreasing from 143.15 to 40.17 mg C g^−1^. These findings demonstrate that cation modification influenced both AHS retention and the interfacial properties of MON, highlighting the potential of biomass-derived AHS-mineral composites for further investigation in carbon management and circular bioeconomy applications.

## Abstract 2

Interactions between biomass-derived artificial humic substances and clay minerals govern organo-mineral associations and influence organic carbon retention.

## Graphical abstract

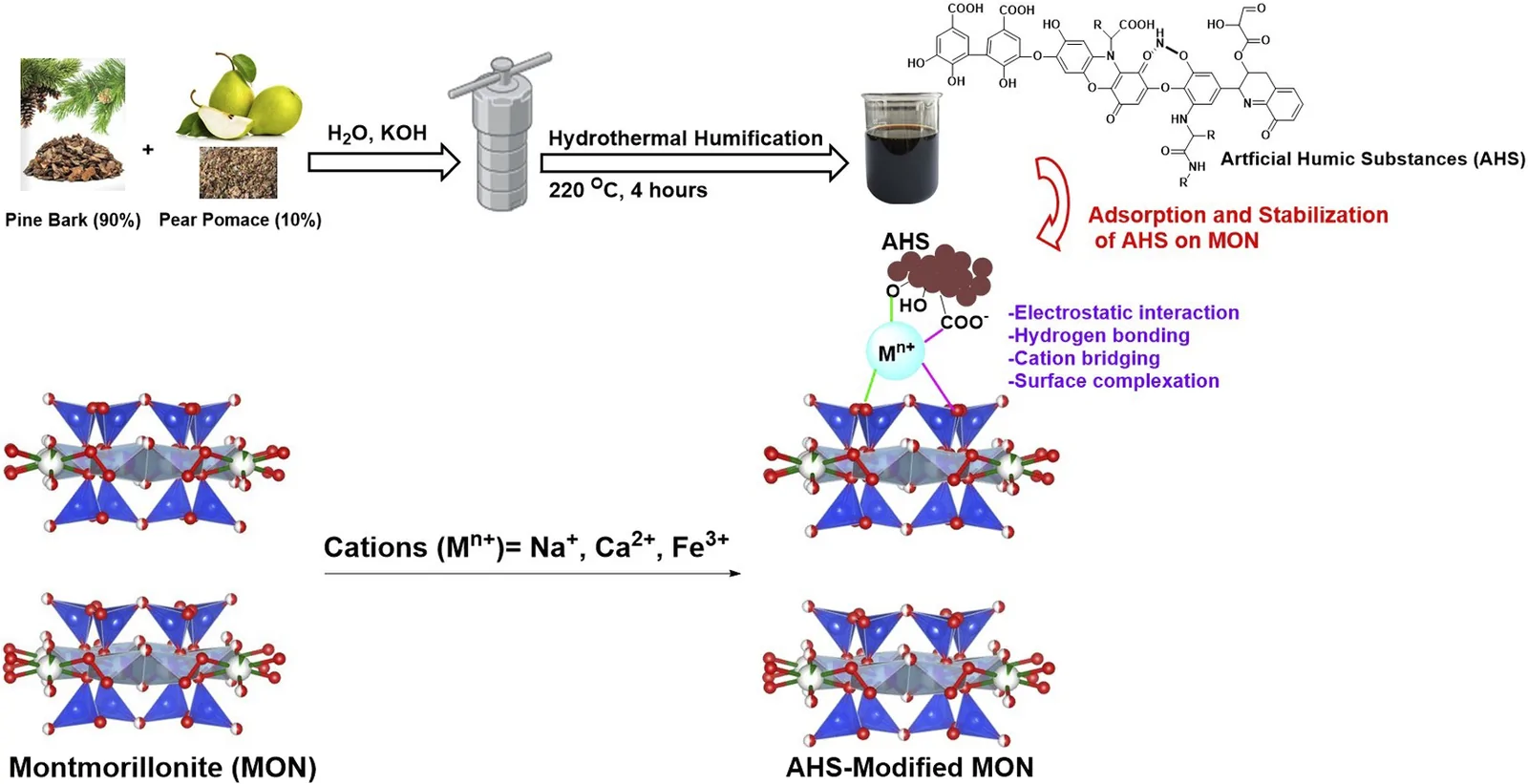

## 1. Introduction

The stabilization of soil organic carbon within mineral matrices is central to terrestrial carbon cycling and climate regulation.^1^ In this context, stabilization encompasses the establishment of persistent organo-mineral associations that enhance organic carbon retention by limiting desorption, remobilization, and subsequent loss from the mineral matrix. Accordingly, land-management strategies that enhance soil carbon storage are widely considered important for mitigating increases in atmospheric CO_2_ levels.^2,3^ Enhancing organic carbon stabilization is important for maintaining soil ecosystem functioning and improving the long-term resilience of terrestrial carbon reservoirs.^4,5^ It is essential to have a clear understanding of the processes governing the long-term stabilization of organic matter in soils.^6^ This process is driven not only by organic inputs but also largely by organo-mineral interactions at the micro- and nanoscale.^7^

Soil structure forms as mineral particles assemble into graded micro- and macro-scale aggregates, creating pore networks that control water retention, solute movement, gas exchange, and microbial access, thereby influencing overall soil function.^8,9^ Among the key stabilization mechanisms, the sorption of organic matter onto reactive mineral surfaces plays a major role. Interactions with clay minerals can reduce microbial decomposition, influence the stability of organic compounds, contribute to soil carbon sequestration, and affect the transport of nutrients and contaminants in soil-water systems.^10,11^

Humic substances (HSs), which can comprise 60–80% of total soil organic matter, are among the most chemically diverse and functionally important fractions of terrestrial organic carbon.^12–14^ These heterogeneous macromolecular assemblies are rich in oxygen-containing functional groups, such as carboxylates, phenolics, carbonyls, quinones, and methoxy groups, which provide metal-binding capacity, redox activity, and amphiphilic characteristics.^15^ Although HSs were traditionally considered refractory end products of microbial transformation, they can exhibit substantial resistance to degradation when stabilized through association with mineral surfaces.^16^ Importantly, their persistence can decrease after detachment from minerals, highlighting the role of organo-mineral interactions in carbon retention over longer timescales.^17–19^

Natural humic substance (NHS) formation occurs over long timescales through interconnected biotic and abiotic processes.^20^ In contrast, hydrothermal humification (HTH) enables the production of artificial humic substances (AHS) under controlled alkaline conditions (180–250 °C, 1–6 h), providing a rapid, reproducible, and tunable alternative to NHS formation.^21,22^ AHS produced *via* HTH possess structural motifs and functional groups, including aromatic domains and carboxyl, phenolic, and quinone functionalities, that are also characteristic of NHS. These similarities may allow AHS to perform soil functions such as carbon stabilization, nutrient retention, and pollutant complexation.^12^ Functionally, these materials have been shown to enhance soil carbon stabilization and aggregate formation,^23–25^ improve nutrient retention through metal chelation (*e.g.*, Fe^3+^, Ca^2+^, and trace micronutrients),^21,26^ and immobilize organic and inorganic pollutants through complexation and adsorption.^27,28^ Nevertheless, similarities in functional-group composition do not necessarily imply structural equivalence between AHS and NHS, and the extent to which these functions are expressed depends on their chemical composition and environmental conditions. In addition to their role in soil systems, AHS are increasingly being investigated for water-treatment applications because of their adsorption capacity and coordination properties.^27,29–31^ This multifunctionality highlights the potential relevance of AHS to integrated soil and water management strategies.

Despite increasing interest in HTH, AHS have been investigated in a limited number of studies, which have primarily focused on their synthesis and basic structural characterization.^32–36^ Their interfacial behavior with common soil minerals and the molecular-scale mechanisms governing these interactions remain poorly understood. A mechanistic understanding of AHS stabilization requires examining interactions between AHSs and expandable phyllosilicate minerals.^37,38^ Montmorillonite (MON), a widely distributed 2 : 1 dioctahedral smectite, is frequently used as a model mineral.^39,40^ Its crystal structure, consisting of tetrahedral–octahedral–tetrahedral layers with permanent structural charges balanced by interlayer cations, confers a high cation-exchange capacity and surface reactivity, making MON a key mediator of organo-mineral interactions.^9,18,41,42^

The type and valence state of exchangeable cations play a major role in determining clay surface chemistry, interlayer structure, and sorption behavior.^9,43,44^ Sodium-saturated MON generally presents greater swelling and increased interlayer accessibility, which may facilitate the diffusion of organic macromolecules.^45^ In contrast, calcium ions can promote electrostatic bridging between negatively charged siloxane surfaces and humic polyanions, enhancing adsorption through outer-sphere complexation.^2^ Ferric ions may provide additional coordination pathways, including the formation of inner-sphere complexes and ligand-exchange reactions with oxygen-containing functional groups of HSs.^40,46^ As a result, cation modification may influence not only adsorption affinity but also the conformational organization, stability, and reversibility of humic layers on mineral surfaces.^2,47–50^

In addition, the adsorption of HSs can modify the physicochemical properties of mineral surfaces.^5^ The accumulation of polyanionic macromolecules may cover reactive aluminum- and iron-hydroxyl edge sites, leading to a more uniform surface-charge distribution and an overall negative electrokinetic potential due to acidic functional groups extending into the surrounding solution.^51,52^ This reorganization of the mineral surface can affect colloidal stability, aggregation behavior, and interparticle interactions.^53–55^ These modifications may influence carbon sequestration dynamics and the mobility and transport of contaminants in soil-water systems.^56–59^

Despite extensive knowledge of interactions between organic matter and mineral phases, less is known about the adsorption and retention of biomass-derived AHS by clay minerals and how these processes are influenced by exchangeable cations. In this study, AHS were produced through hydrothermal humification of pear pomace and pine bark, representing agro-industrial and woody biomass residues, respectively. Their interactions with pristine and Na^+^-, Ca^2+^-, and Fe^3+^-modified MON were then investigated. The selected cations differ in chemical identity and charge, allowing their effects on AHS adsorption and retention to be compared without attributing the observed differences solely to cation valence. Adsorption and desorption experiments, combined with structural and surface characterization, were used to examine how cation modification affects AHS-MON interactions and the retention of AHS within the resulting organo-mineral composites. The findings provide insight into the formation and stability of biomass-derived organic matter-mineral associations and their potential to enhance carbon retention. However, their effectiveness for long-term carbon sequestration and soil applications requires further evaluation under environmentally relevant conditions.

## 2. Materials and methods

### 2.1. Experimental design

The experimental design comprised three interconnected stages: production of biomass-derived AHS, modification of MON, and evaluation of AHS adsorption and retention by pristine and modified MON (Fig. 1). AHS were produced through HTH of a pear pomace-pine bark mixture, selected to represent agro-industrial and woody biomass residues, respectively. In parallel, MON was separately modified with Na^+^, Ca^2+^, and Fe^3+^ to obtain Na-MON, Ca-MON, and Fe-MON. The influence of MON modification on AHS adsorption was evaluated across a range of initial AHS concentrations, followed by desorption experiments to assess the release of adsorbed AHS under the conditions tested. Residual and desorbed AHS in the liquid phase were quantified by total organic carbon (TOC) analysis. Structural, textural, thermal, and spectroscopic techniques were subsequently used to characterize AHS, the mineral materials, and the resulting organo-mineral complexes and to evaluate physicochemical changes associated with MON modification and AHS adsorption.

**Fig. 1:**
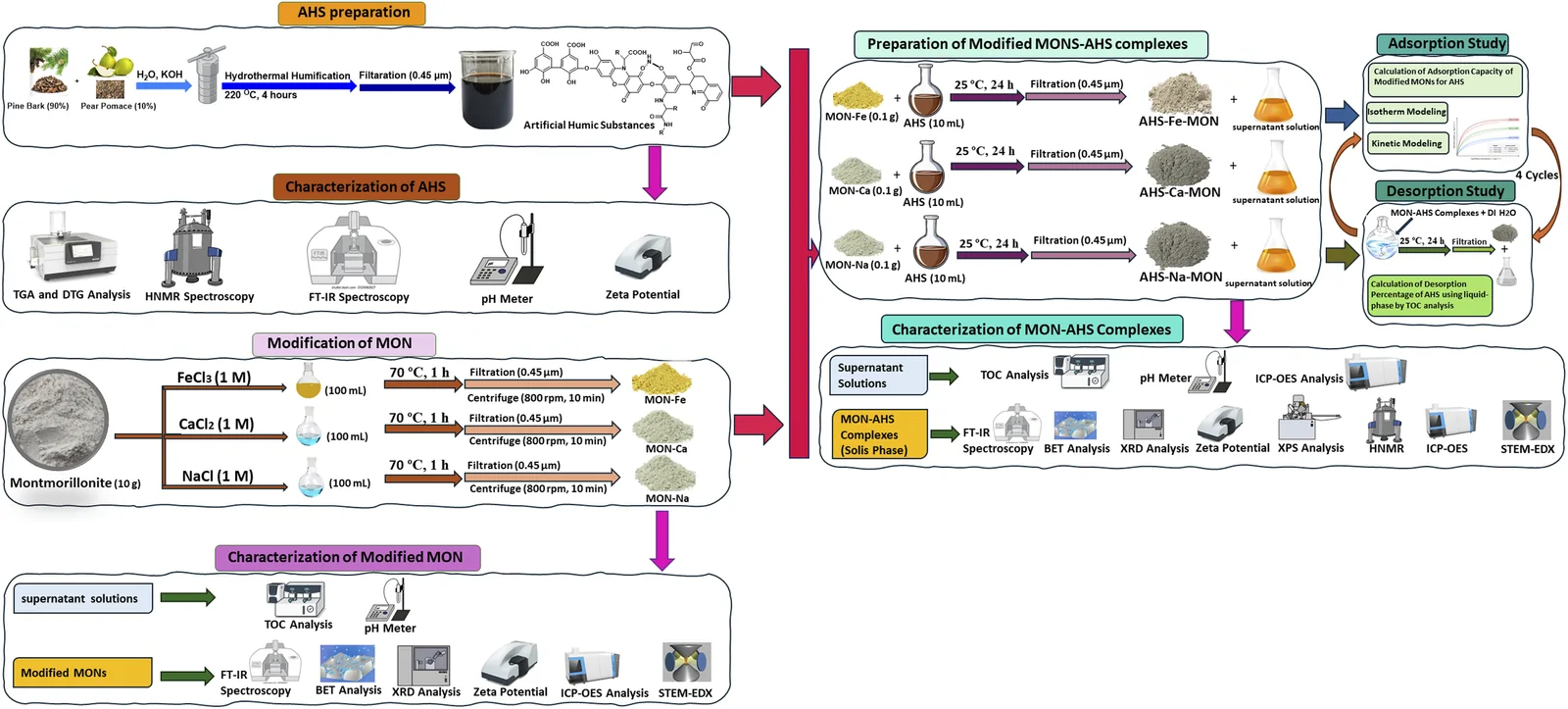
Schematic overview of the experimental design, including AHS production, cation modification of montmorillonite, preparation of AHS-modified MON, and subsequent adsorption, desorption, and material characterization.

### 2.2. Preparation of cation-modified montmorillonite

Montmorillonite (MON), NaCl, CaCl_2_, and FeCl_3_ (extra pure, 99%) were purchased from Sigma-Aldrich (Merck KGaA, Germany). Oxalic acid (anhydrous, 98%) and ammonium oxalate monohydrate (ACS reagent grade, 99.0–101.0%) were obtained from Alfa Aesar (Germany), while sodium dithionite was supplied by Fisher Scientific (Germany). Sodium bicarbonate (ACS reagent grade, ≥99.7%), sodium citrate dihydrate (≥99%), hexamminecobalt(iii) chloride, and potassium chloride (≥99.5%) were obtained from Sigma-Aldrich (Merck KGaA, Germany). All chemicals were used as received, without further purification. Deionized water was used for the preparation of all aqueous solutions.

MON was subjected to cation modification using aqueous solutions of NaCl, CaCl_2_, and FeCl_3_. 10 g of MON was dispersed separately in 100 mL of 1 M NaCl, CaCl_2_, or FeCl_3_ solution and stirred continuously at 70 °C for 1 h to facilitate cation incorporation into the MON through ion exchange and surface complexation.^60^ The suspensions were subsequently filtered through 0.45 µm membranes (Whatman), and the recovered solids were washed three times with 10 mL of deionized water per wash by centrifugation at 8000 rpm for 10 min (Hettich Universal 320, Germany) to remove excess electrolyte and unbound ions. The resulting cation-modified MON samples, designated MON-Na, MON-Ca, and MON-Fe, were dried at 40 °C for 24 h and stored under ambient conditions until further use.

### 2.3. Hydrothermal humification of biomass and preparation of MON-AHS complexes

The biomass feedstock consisted of a mixture of pear pomace generated during juice production (85% moisture content) and pine bark (50% moisture content). Pear pomace and pine bark were selected as representative agro-industrial and woody biomass residues, respectively, as compositionally distinct lignocellulosic feedstocks for hydrothermal humification. The feedstocks were mixed at a ratio of 10 : 90 on a wet-weight basis. Potassium hydroxide (KOH, ≥85%, Merck) was added at 20 wt% on a dry biomass basis. This KOH loading was selected based on preliminary experiments to achieve a final pH of 7.0. Deionized water was added to achieve a biomass-to-water ratio of 0.3 (w/w). The resulting slurry was transferred into 45 mL pressure vessels (Parr Instrument Company, Moline, IL, USA) and heated at 220 °C for 4 h in a ventilated oven. The residence time was measured from the moment the oven reached the set temperature; the heating time was approximately 25 min. After the reaction, the suspension was cooled to room temperature and filtered through a 0.45 µm membrane filter. The resulting hydrothermal filtrate was used as AHS without further purification; therefore, it may contain both humic-like components and smaller dissolved organic compounds generated during hydrothermal conversion. The liquid phase, containing the produced AHS, was collected and stored for the preparation of MON-AHS complexes.

MON-AHS complexes were prepared by mixing 0.1 g of pristine or cation-modified MON (MON-Na, MON-Ca, or MON-Fe) with 10 mL of AHS solution at concentrations ranging from 0.5 to 25 g L^−1^ at 25 °C for 24 h.^61^ For kinetic experiments, 0.1 g of pristine or modified MON were mixed with 10 mL of AHS at a concentration of 25 g L^−1^, and the contact times was varied from 1 to 48 h. At the end of each experiment, the suspensions were filtered through a 0.45 µm membrane filter, and the recovered solid phases were dried at 40 °C for further analysis. The liquid phases collected after adsorption experiments were also analyzed by inductively coupled plasma optical emission spectrometry (ICP-OES) to quantify dissolved elements and further investigate cation release and redistribution during AHS-MON interactions.

### 2.4. Chemical extraction procedures for Fe-MON and pristine MON

To investigate the distribution of Fe species in pristine MON and Fe-modified MON (Fe-MON), complementary chemical extraction procedures were performed prior to ICP-OES analysis. These procedures were selected to assess operationally defined Fe fractions associated with cation exchange, acid-labile species, poorly crystalline Fe-bearing phases, and reducible Fe oxides. Each extraction was conducted on a separate subsample to avoid interference between extraction reagents and to reduce the risk of interpreting overlapping extraction fractions as discrete, additive Fe pools.

Exchangeable cations were investigated using unbuffered hexamminecobalt(iii) chloride extraction following the general principles of ISO 23470:2018.^62^ The concentrations of Fe and other relevant cations in the extracts were determined by ICP-OES. The method was used to assess the Fe released under cation-exchange conditions; however, the extracted Fe concentration was not assumed to represent exclusively exchangeable Fe^3+^, because Fe hydrolysis and potential reprecipitation during extraction may affect its recovery.

To obtain an operational upper-bound estimate of exchangeable Fe, a separate extraction was performed using 1 M KCl acidified to pH 2.5 for 15–30 min. The Fe and Al concentrations in the resulting extracts were determined by ICP-OES.^63^ Because the acidic conditions may promote the dissolution of labile hydrolyzed Fe species in addition to the displacement of exchangeable cations, the Fe recovered by this procedure was interpreted as acid-extractable Fe rather than as a selective measure of exchangeable Fe.

Poorly crystalline Fe-bearing phases were assessed by extraction with 0.2 M acid ammonium oxalate at pH 3 for 2 h in the dark, following Schwertmann (1964).^64^ The concentrations of Fe, Al, and Si in the extracts were determined by ICP-OES. Oxalate-extractable Fe was interpreted operationally as an indicator of Fe associated with poorly crystalline and short-range-order phases, while recognizing that the extraction may also dissolve other reactive or nanocrystalline Fe-bearing phases.

Reducible Fe-bearing phases were further investigated using the dithionite–citrate–bicarbonate (DCB) extraction at 80 °C, following the procedure of Mehra and Jackson (1958).^65^ The concentrations of Fe, Al, and Si in the extracts were determined by ICP-OES. DCB-extractable Fe was used to estimate the pool of Fe susceptible to reductive extraction under the specified conditions. Because chemical extraction is operationally defined rather than absolutely selective for specific mineral phases, the DCB results were not interpreted as direct evidence for a particular Fe mineral or oxidation state.

All Fe concentrations were expressed relative to the corresponding dry sample mass, using the measured extract concentrations, extraction volumes, dilution factors, and sample masses. Where appropriate, the results for Fe-MON were compared with those for pristine MON to assess the contribution of Fe associated with the modification treatment. The extraction fractions were evaluated comparatively rather than summed, because the extractants may dissolve overlapping Fe pools. Accordingly, the results were used to assess the operationally defined fractions of exchangeable, acid-labile, oxalate-extractable, and DCB-extractable Fe, rather than to establish a definitive mineralogical partitioning of total Fe.

### 2.5. Adsorption and desorption experiments

Adsorption isotherm experiments were conducted under the same conditions as those used to prepare the MON-AHS complexes. After equilibrium was reached, the samples were filtered through a 0.45 µm membrane filter, and the TOC concentration of the filtrate was measured by TOC analysis (Shimadzu, Japan). The amount of adsorbed organic carbon was calculated by mass balance. The decrease in TOC concentration in the filtrate was used to quantify the apparent removal of organic carbon from the aqueous phase. However, because TOC analysis measures total organic carbon rather than individual AHS components, the observed decrease cannot be attributed exclusively to the adsorption of humic-like substances onto the clay. The adsorption capacity of MON samples was calculated according to eqn (1):1
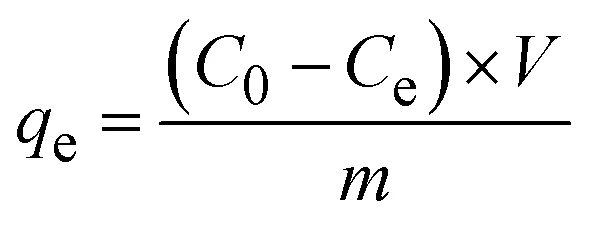
where *q*_e_ (mg C per g clay) is the adsorption capacity at equilibrium, *C*_0_ and *C*_e_ (mg C L^−1^) are the initial and equilibrium TOC concentrations, respectively, *V* is the volume of the solution (L), and *m* is the mass of MON (g).

The adsorption data were fitted using the Langmuir (eqn (2)), Freundlich (eqn (3)), and Sips (eqn (4)) isotherm models. These models were fitted to the experimental data as described above.2
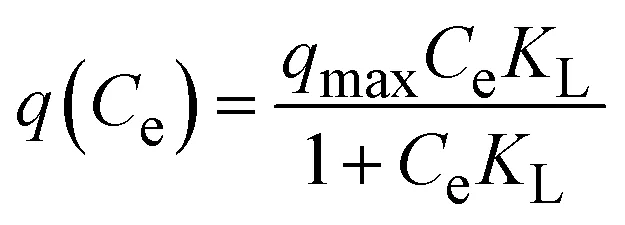
3
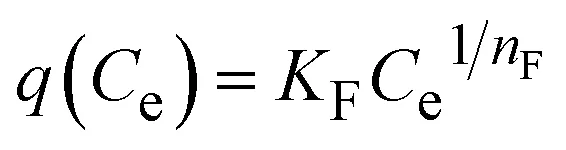
4
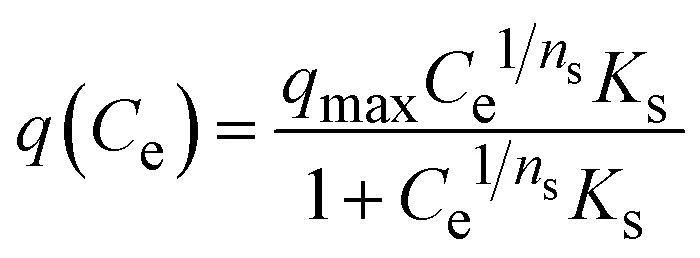
where *q*_max_ (mg C per g clay) is the maximum adsorption capacity, *K*_L_ (L per mg per C) is the Langmuir adsorption constant, *K*_F_ ((mg C per g clay)(L per mg per C)^1/*n*_F_^) is the Freundlich adsorption constant, and *n*_F_ is the Freundlich heterogeneity factor. In the stated Sips equation, *K*_S_ (L per mg per C)^1/*n*_s_^ is the Sips adsorption constant, and *n*_s_ is the Sips model exponent constant.

The adsorption kinetics of AHS onto Na-, Ca-, and Fe-modified MON were investigated by monitoring adsorption capacity as a function of contact time. The adsorption capacity was calculated from the change in dissolved organic carbon concentration, as measured by TOC analysis. The experimental kinetic data were fitted to the pseudo-first-order (PFO) and pseudo-second-order (PSO) models. The PFO model was expressed as follows:5
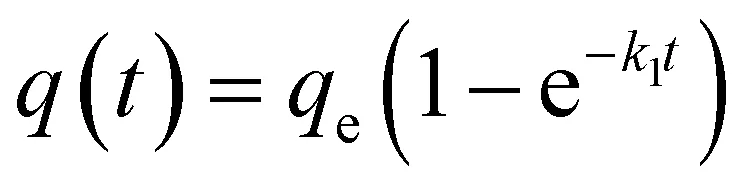
where *q*_e_ is the fitted equilibrium adsorption capacity (mg C per g clay) and *k*_1_ is the PFO rate constant (1/h). The PSO model was expressed as follows:6
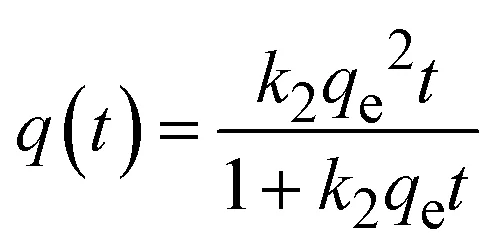
where *k*_2_ is the PSO rate constant (g clay per mg per C h). The kinetic parameters were obtained by nonlinear regression of the experimental adsorption capacities as a function of contact time.

Desorption experiments were performed over four consecutive adsorption–desorption cycles using the recovered MON-AHS complexes. After adsorption, the solids were redispersed in 10 mL of deionized water and shaken at 25 °C for 24 h. The suspensions were then filtered through a 0.45 µm membrane filter, and the TOC concentration in the filtrates was measured to quantify the organic carbon released during each desorption step. The recovered solids were then used in the next adsorption cycle. This procedure was repeated over four consecutive cycles to evaluate changes in adsorption capacity and the release of AHS-derived organic carbon from Na-MON, Ca-MON, and Fe-MON.

### 2.6. Material characterization

AHS, pristine MON, cation-modified MON (MON-Na, MON-Ca, and MON-Fe), and the corresponding MON-AHS complexes were characterized using structural, textural, thermal, and spectroscopic techniques, as described below.

The elemental composition of AHS was determined using a Vario MICRO cube CHNOS analyzer (Elementar Analysensysteme GmbH, Langenselbold, Germany). Carbon, hydrogen, and nitrogen contents were quantified using a thermal conductivity detector (TCD), whereas oxygen content was calculated by difference. The elemental composition of the solid and liquid samples was further analyzed by inductively coupled plasma-optical emission spectrometry (ICP-OES; Optima 8000, PerkinElmer, Waltham, MA, USA).

Thermogravimetric analysis (TGA) was performed using a TG 209 F1 Libra analyzer (NETZSCH, Germany) under a high-purity N_2_ atmosphere (20 mL min^−1^). Samples were heated from 25 to 900 °C at a rate of 10 °C min^−1^. The extent of conversion was calculated as *x*(*t*) = (*m*_0_ − *m*_*t*_)/(*m*_0_ − *m*_f_), where *m*_0_ is the initial dry mass, *m*_*t*_ is the mass at time *t*, and *m*_f_ is the residual mass at 900 °C. Derivative thermogravimetric (DTG) curves were obtained from the first derivative of the conversion profile with respect to temperature. The residual mass fraction (TGA_res_) was calculated as *m*_f_/*m*_0_.

^1^H NMR analyses were performed on the initial AHS solution and on the liquid phase recovered after adsorption experiments. The samples after freeze-drying were dissolved in D_2_O and analyzed using an Agilent 400 MHz spectrometer. The ^1^H NMR spectra were recorded with a relaxation delay of 1 s and 128 scans. The spectra were processed using MestReNova software. The residual solvent signal from D_2_O (4.8 ppm) was excluded from the analysis. Four spectral regions were integrated: region I (<2 ppm, alkyl protons), region II (2–3 ppm, aliphatic protons), region III (3–4.3 ppm, oxygenated aliphatic protons), and region IV (5–7.5 ppm, aromatic, phenolic, and furfural-derived protons).

The crystal structure of the samples was characterized by X-ray diffraction (XRD, Rigaku Ultima IV, Tokyo, Japan) using Cu Kα radiation operated at 40 kV and 40 mA. Diffractograms were recorded over a 2*θ* range of 5–80°. Phase identification was analyzed using Match 3 software and the ICDD PDF-4+ crystallographic database (database version 4.2103). Surface morphology was examined using a scanning electron microscope (SEM, HITACHI S-4800, Tokyo, Japan). Scanning transmission electron microscopy (STEM) analyses were performed using a double aberration-corrected JEOL JEM-ARM200F transmission electron microscope operated at an accelerating voltage of 80 kV. The instrument was equipped with a cold-field emission gun, a high-angle silicon drift energy-dispersive X-ray (EDX) detector with a solid angle of up to 0.98 sr and a detection area of 100 mm^2^, and a Gatan Quantum post-column electron energy-loss spectroscopy (EELS) system.

FTIR spectra were collected on a Nicolet iS 5 spectrometer equipped with an iD5 attenuated total reflectance (ATR) unit (Thermo Fisher Scientific) over the wavenumber range of 4000–400 cm^−1^, at a resolution of 2 cm^−1^, with 32 scans each for the sample and background. Surface charge properties were evaluated by measuring the zeta potential using a Zetasizer ZS (Malvern Instruments, UK). Zeta potential measurements were performed before and after AHS adsorption using Milli-Q water as the dispersion medium. Three replicates were tested for each sample, with at least six measurements per replicate at 2 min intervals. The pH values of pristine and cation-modified MON samples, before and after AHS adsorption, were measured using a Mettler Toledo FiveEasy F20 pH meter (Mettler Toledo GmbH, Switzerland).

The specific surface area, pore volume, and pore size distribution of pristine, cation-modified, and AHS-modified MON samples were determined by N_2_ physisorption at 77 K using the Brunauer–Emmett–Teller (BET) method (Quantachrome Instruments, USA). Organic and inorganic carbon contents were quantified using an automatic carbon analyzer (TOC-L, Shimadzu, Japan). Total organic carbon (TOC) was calculated as the difference between total carbon and inorganic carbon.

Surface chemical composition was investigated by X-ray photoelectron spectroscopy (XPS, Kratos Axis Supra+, UK) using monochromated Al Kα radiation at 15 kV and 20 mA. Spectra were processed using CasaXPS software, and peak fitting was performed using GL(30) line shapes, comprising 70% Gaussian and 30% Lorentzian contributions.

### 2.7. Statistical analysis

All adsorption experiments involving AHS and clay were performed in triplicate, and the results are expressed as the mean ± standard deviation (SD). Error bars represent the SD of the three replicate measurements. The Langmuir, Freundlich, and Sips adsorption isotherm models, as well as the adsorption kinetic models, were fitted to the experimental data using nonlinear least-squares regression in Origin 2023 (OriginLab Corporation, Northampton, MA, USA).

## 3. Results and discussion

### 3.1. Properties of AHS

The synthesized AHS are dark-colored, heterogeneous organic materials derived from biomass. They exhibit several compositional and functional characteristics associated with the natural counterpart (NHS).

As shown in Table S1, AHS have an H/C ratio of 1.52 and an O/C ratio of 0.91, consistent with previous studies on AHS.^12^ The DTG profile (Fig. 2a) shows three main decomposition peaks centred at 170, 245, and 435 °C. The peak at approximately 170 °C may be attributed to the decomposition of low-molecular-weight compounds. The peak at around 245 °C may be associated with the decomposition of less thermally stable, oxygenated structures and weakly condensed biomass-derived fragments, including oxygen-rich aliphatic structures and carboxyl-containing groups. The higher-temperature peak near 435 °C can be associated with a thermally stable fraction, potentially including more condensed organic structures.

**Fig. 2:**
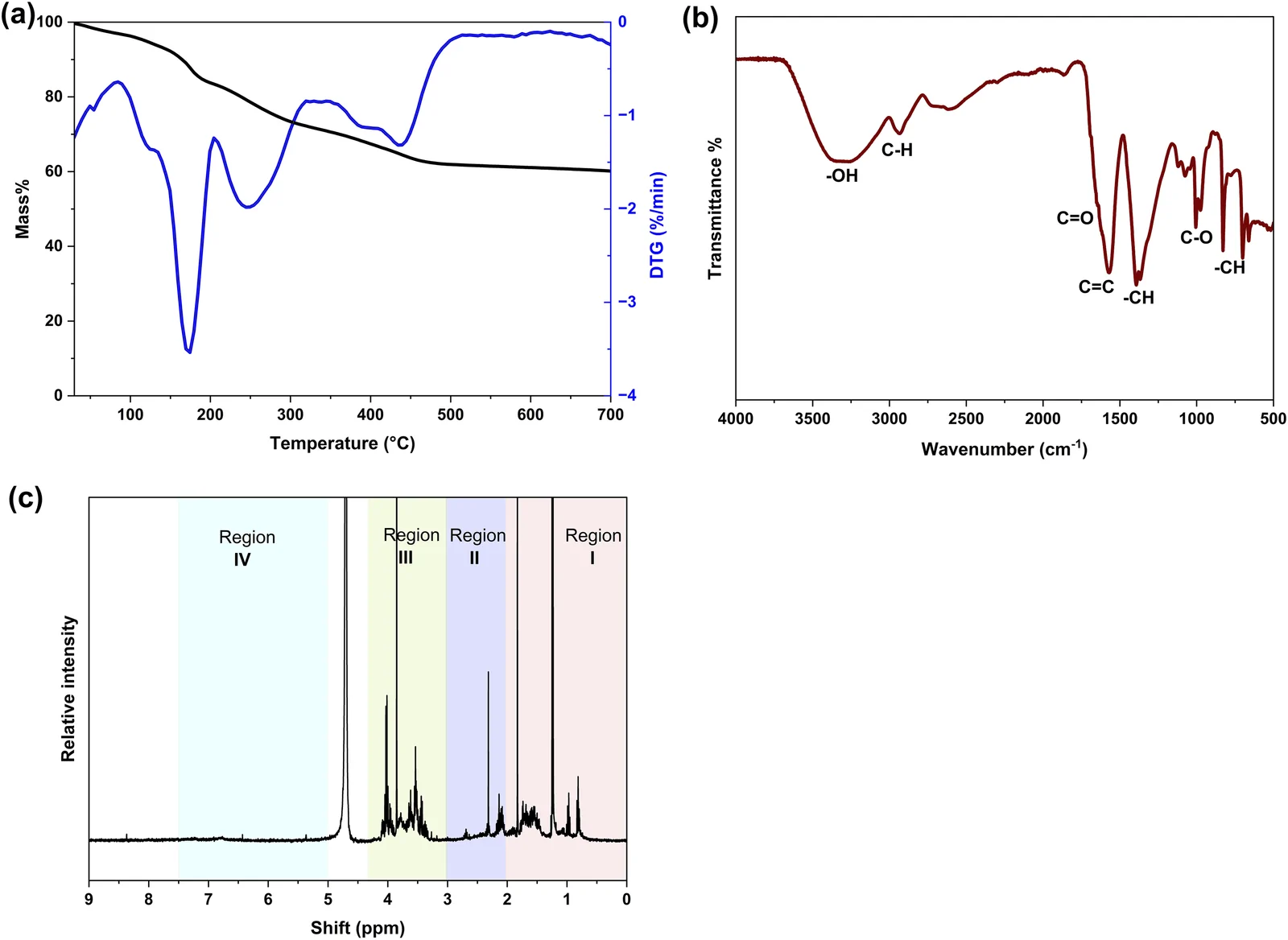
Structural properties of AHS: (a) DTG and TGA curves under an inert N_2_ atmosphere, (b) FT-IR spectrum, and (c) ^1^H NMR spectrum acquired in D_2_O (solvent signal at 4.8 ppm).

The FTIR spectrum (Fig. 2b) shows the presence of various oxygen-containing functional groups in AHS. The broad absorption band in the 3000–3500 cm^−1^ region is attributed to O–H stretching vibrations of hydroxyl groups. Bands in the 2800–3000 cm^−1^ region are assigned to aliphatic C–H stretching vibrations. The band at approximately 1700 cm^−1^ is attributed to C=O stretching vibrations, consistent with the presence of carboxylic acid groups. The bands around 1570 cm^−1^ may be associated with C=C stretching vibrations in aromatic and conjugated structures. Signals in the 1368–1394 cm^−1^ region may arise from carboxylate groups and aliphatic C–H vibrations, while bands in the 970–1000 cm^−1^ region are associated with C–O stretching vibrations of oxygen-containing functional groups. The signals around 830 and 662–700 cm^−1^ may be attributed to out-of-plane C–H bending vibrations of substituted aromatic rings. Overall, the FTIR spectrum indicates the presence of oxygen-containing functional groups, aliphatic structures, and a contribution from aromatic or conjugated structures in AHS.

The ^1^H NMR spectrum of AHS is shown in Fig. 2c, and the relative signal distributions are summarized in Table S2. Although a few sharp peaks can be identified, the spectra are predominantly broad and poorly resolved, a characteristic feature of heterogeneous humic-like substances.^66^ The spectrum is dominated by region I (0–2 ppm, 47.1%) and region III (3–4.3 ppm, 43.0%), indicating the coexistence of aliphatic and oxygenated proton environments. Region II (2–3 ppm) accounts for a smaller contribution (8.2%) and may be attributed to aliphatic protons adjacent to carbonyl or carboxyl-containing groups. Region IV (5–7.5 ppm) accounts for only 1.7% of the total integrated signal area, suggesting a relatively low contribution from aromatic and phenolic proton environments in the measured dissolved fraction. However, this relative signal contribution does not directly quantify the aromatic-carbon content or the mass fraction of aromatic structures in bulk AHS. Several well-defined resonances can be assigned to low-molecular-weight compounds potentially generated during hydrothermal humification. Signals at 1.24 and 4.03 ppm are attributed to lactic acid, which can form during alkaline hydrothermal conversion of carbohydrates through the retro-aldol cleavage of glucose-derived intermediates. The peak at 1.82 ppm is assigned to acetic acid, a possible product of carbohydrate degradation during hydrothermal processing. The signal at 2.30 ppm is consistent with levulinic acid, another degradation product forming from carbohydrate. The signal at 3.85 ppm is assigned to methoxyl protons associated with lignin-derived structures. Finally, weak signals at approximately 5.35 and 6.40 ppm may indicate minor contribution from unsaturated and aromatic or phenolic proton environments, respectively.

Overall, the NMR results indicate that the analyzed dissolved AHS fraction contains substantial aliphatic and oxygenated proton contributions, together with a smaller contribution from aromatic proton environments.^67^ These structural characteristics may influence interactions with mineral surfaces and could be relevant to potential applications in soil amendment and environmental remediation.

### 3.2. Properties of pristine and cation-modified montmorillonite

Pristine MON was modified with Na^+^, Ca^2+^, and Fe^3+^ to investigate the influence of cation modification on AHS adsorption.

The FTIR spectra of pristine and cation-modified MON (Fig. 3a) illustrate characteristic adsorption bands of the clay mineral. The Al–OH stretching bond at approximately 3620 cm^−1^ remains essentially unchanged after modification, suggesting that the local environment of structural hydroxyl groups is largely preserved. Slight differences in the O–H stretching region (approximately 3400 cm^−1^) and around 1630–1640 cm^−1^ may reflect differences in the hydration states of the exchangeable cations; smectite clays can retain water associated with interlayer cations even after drying.^68^ The Si–O stretching bands at approximately 1206, 1004, and 973 cm^−1^, together with the bands around 797 and 520 cm^−1^ assigned to Si–O–Si vibrations, are present in all samples, indicating that the characteristic aluminosilicate framework vibrations are retained after cation modification.^69^ A slight increase in the intensity of the structural Al–OH band after modification may reflect changes in the local environment; however, this difference alone does not establish a specific structural change. Overall, the absence of significant band shifts or new absorption bands suggests cation modification does not cause major detectable changes in the characteristic FTIR features of MON.

**Fig. 3:**
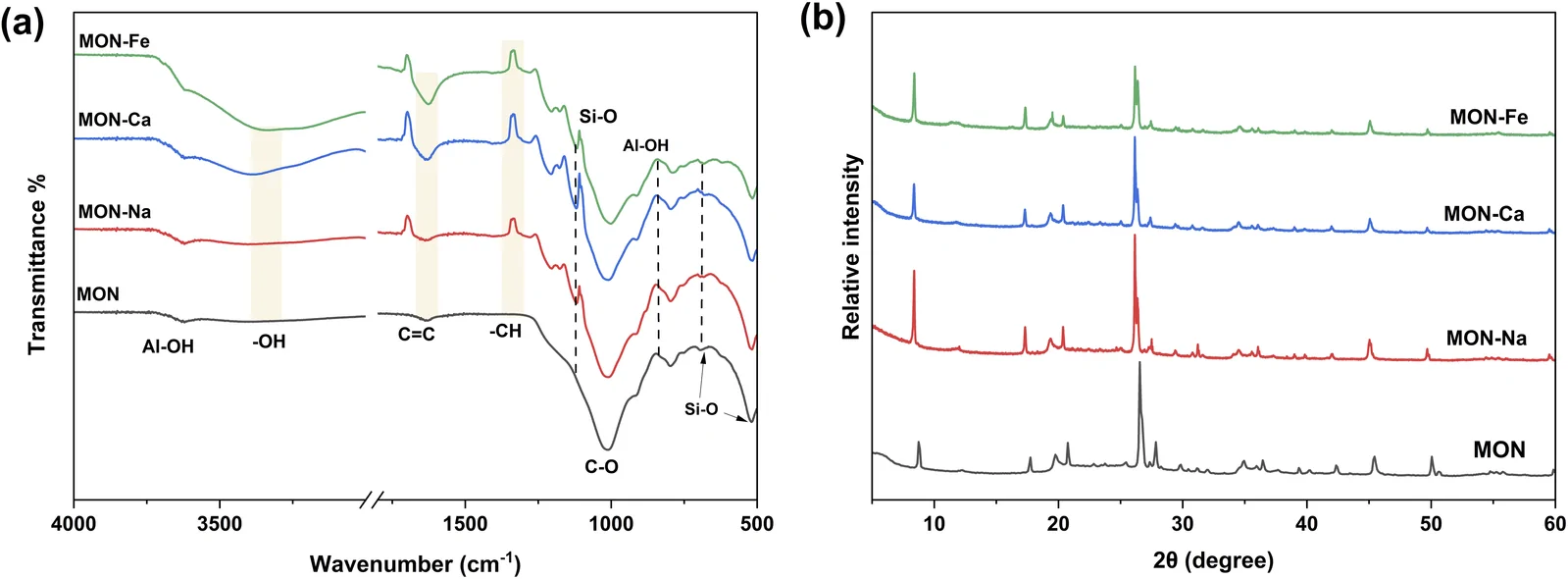
(a) FTIR spectra and (b) XRD patterns of pristine MON and cation-modified samples (MON-Na, MON-Ca, and MON-Fe).

Zeta potential and pH measurements showed systematic changes after cation modification (Table 1). Pristine MON exhibits a negative zeta potential (−34.3 mV), consistent with the permanent negative charge of smectite layers. MON-Na remains negatively charged (−21.5 mV), whereas Ca-MON exhibits less negative values (−8.5 mV), and MON-Fe represents a positive value (+27.0 mV). These changes indicate that cation modification alters the electrokinetic properties of the mineral. Simultaneously, the pH decreased from 4.04 for pristine MON to 2.89, 2.96, and 1.37 for MON-Na, MON-Ca, and MON-Fe, respectively. However, the trend cannot be attributed solely to increasing cation valence, as the measured zeta potential also depends on cation-specific interactions, surface chemistry, pH, and the composition of the surrounding solution.^37^

**Table 1:** BET surface area, pore characteristics, zeta potential, and pH of pristine, cation-modified, and MON-AHS samples

Sample	Surface area (m^2^ g^−1^)	Pore width (nm)	Pore volume (cm^3^ g^−1^)	Zeta potential (mV)	pH
AHS	—	—	—	−47	8.31
MON	230.0	6.0	0.37	−34.3	4.04
MON-Na	216.4	6.0	0.34	−21.5	2.89
MON-Ca	128.2	6.0	0.27	−8.5	2.96
MON-Fe	215.9	6.0	0.38	27.0	1.37
MON-AHS	125.1	0.82	0.04	−37.4	7.86
MON-Na-AHS	58.5	10.1	0.19	−27.5	8.03
MON-Ca-AHS	43.9	9.4	0.17	−28.7	8.01
MON-Fe-AHS	46.2	6.0	0.14	−30.1	7.46

BET analysis revealed differences in the textural properties of pristine and cation-modified MON (Table 1). Pristine MON exhibited a surface area of 230.0 m^2^ g^−1^, comparable to those of Na-MON (216.4 m^2^ g^−1^) and Fe-MON (215.9 m^2^ g^−1^). In contrast, Ca-MON showed a lower surface area (128.2 m^2^ g^−1^) and a pore volume of 0.27 cm^3^ g^−1^, indicating differences in N_2_-accessible surface area and pore characteristics. These differences may reflect changes in particle aggregation, interparticle voids, or pore accessibility associated with cation modification. The relatively high surface area of Fe-MON compared with Ca-MON indicates that textural properties differ between these materials; however, possible interactions between Fe-associated surface species and oxygen-containing functional groups of AHS may also influence adsorption behavior.^70^ The average pore widths of the pristine and cation-modified samples were approximately 6.0 nm, within the mesopore size range. Overall, the BET results indicate that cation modification influences the N_2_-accessible surface area and pore characteristics of MON. The differences in AHS retention among the modified clays suggest that textural properties alone may not fully explain the observed adsorption behavior. Cation-dependent surface chemistry may also contribute to these differences, although the relative contributions of surface accessibility, electrostatic interactions, and possible cation-mediated interactions cannot be conclusively distinguished from the present results.^37,40^

The XRD patterns (Fig. 3b) indicate characteristic reflections of MON, including the basal (001) reflection and higher-angle reflections at approximately 17.8°, 19–20°, and 45.5°, associated with the layered silicate structure. A prominent reflection at approximately 26.2° is attributed to quartz, a common impurity in natural clays. The basal (001) reflection occurs at approximately 8.8° (2*θ*) for pristine MON and shifts to around 8.3–8.4° after cation modification. The basal spacing *d*_001_ was calculated from the position of the (001) reflection using Bragg's law:

7 *nλ* = 2*d* sin *θ*

where *n* = 1 for first-order diffraction, *λ* = 1.540 Å for Cu Kα radiation, *d* is the basal spacing, and *θ* is half of the measured diffraction angle. According to Bragg's law, basal spacings were approximately 10.05 Å for pristine MON and 10.52–10.54 Å for the modified samples. However, the quartz reflection also exhibits a shift, and instrumental contributions to the observed peak-position differences cannot be excluded. In the absence of an independent calibration or replicate XRD measurements, these calculated values should therefore be regarded as estimates based on the observed peak positions rather than conclusive evidence of a change in interlayer spacing. Accordingly, the XRD data do not establish whether cation modification caused a genuine change in basal spacing or identify the mechanism underlying the observed peak shifts.

Overall, the XRD, FTIR, and SEM results suggest that the principal structural features and overall morphology of MON remain largely preserved following cation modification and AHS treatment, although subtle structural changes cannot be excluded.

### 3.3. Chemical extraction of iron from pristine MON and Fe-MON

To further investigate the operationally defined Fe pools in pristine MON and Fe-modified MON (Fe-MON), parallel extractions were performed using hexamminecobalt(iii) chloride, acidified KCl, acid ammonium oxalate, and dithionite–citrate–bicarbonate (DCB), followed by ICP-OES analysis (Table S8). These procedures probe Fe released under different chemical conditions; however, the extracted Fe fractions are operationally defined and should not be interpreted as uniquely identified mineralogical or oxidation-state-specific species.

The extraction analyses revealed markedly greater Fe recoveries from Fe-MON than from pristine MON across all four procedures. Hexamminecobalt(iii) chloride recovered 59.3 ± 7.4 mg Fe/g from Fe-MON, compared with 1.14 ± 0.06 mg Fe/g from MON, while acidified KCl yielded 62.8 ± 7.0 and 0.215 ± 0.006 mg Fe/g, respectively. Acid ammonium oxalate and dithionite–citrate–bicarbonate (DCB) extraction yielded 54.2 ± 2.5 and 58.6 ± 0.8 mg Fe/g from Fe-MON, compared with 1.32 ± 0.03 and 1.70 ± 0.06 mg Fe/g from MON, respectively. The consistently elevated recoveries from Fe-MON indicate that the Fe introduced during FeCl_3_ treatment is susceptible to mobilization under a range of chemically distinct extraction conditions. Although hexamminecobalt(iii) chloride is conventionally used to assess exchangeable cations, the recovered Fe cannot be assigned exclusively to exchange sites. The substantial recovery under acidified KCl conditions may additionally reflect the mobilization of acid-sensitive Fe species, while oxalate-promoted dissolution and DCB-induced reductive dissolution are consistent with the presence of reactive Fe-bearing phases, potentially including poorly crystalline Fe (oxyhydr)oxides. Nevertheless, because these extraction procedures are operationally defined and may mobilize overlapping Fe fractions, their recoveries cannot be interpreted as selective measurements of discrete Fe pools. Collectively, the results support the presence of a heterogeneous, readily extractable Fe fraction in Fe-MON, including Fe that is not necessarily associated with conventional cation-exchange sites. However, the extraction data alone do not establish the precise speciation, mineralogical identity, or coordination environment of the introduced Fe, nor do they permit its unambiguous quantitative partitioning among distinct Fe-bearing phases.

Collectively, the extraction results indicate that Fe modification introduced or increased a substantial pool of chemically extractable Fe. However, the data do not establish the precise speciation, mineralogical identity, oxidation state, or coordination environment of this Fe, nor do they permit its quantitative partitioning among exchange-associated Fe, hydrolyzed Fe species, and other Fe-bearing phases. Accordingly, the extraction results are interpreted as complementary evidence of Fe mobilization under defined chemical conditions rather than as definitive identification of discrete Fe pools.

### 3.4. Adsorption of AHS onto pristine and cation-modified montmorillonite

TOC analysis of the supernatant solutions was used to quantify residual dissolved organic carbon, from which carbon retention by pristine and cation-modified MON was calculated by mass balance (Table 2). The amount of retained carbon increased from 143 mg C g^−1^ for pristine MON to 316 mg C g^−1^ for Na-MON, 413 mg C g^−1^ for Ca-MON, and 546 mg C g^−1^ for Fe-MON. The percentage of retained carbon followed the same trend, increasing from 6.7% for pristine MON to 25.8% for Fe-MON. These findings demonstrate that cation modification substantially influenced carbon retention, with Fe-MON exhibiting the highest carbon uptake. The differences in adsorption indicate that AHS-mineral interactions depend on the nature of the modifying cation. However, because the investigated cations differ in both chemical identity and charge, the observed trend cannot be attributed exclusively to cation valence. Na^+^ modification may reduce electrostatic repulsion between negatively charged AHS and mineral surfaces, whereas Ca^2+^ may additionally promote cation-mediated associations involving carboxylate groups.^40^ Fe-associated species may interact with oxygen-containing functional groups of AHS, potentially contributing to the highest carbon uptake observed for Fe-MON; however, the present results do not independently establish this mechanism.^37^

**Table 2:** Adsorption of organic carbon from AHS onto pristine and modified MON, determined by TOC analysis. Adsorbed carbon is expressed as mg C per g of clay. The initial TOC concentration of the filtered AHS solution was 21.2 g C L^−1^

Sample	Adsorbed organic carbon (mg C per g clay)	C retained (%)
MON-AHS	143 ± 7.15	6.7 ± 0.34
MON-Na-AHS	316 ± 15.80	14.9 ± 0.75
MON-Ca-AHS	413 ± 20.63	19.5 ± 0.98
MON-Fe-AHS	546 ± 27.30	25.8 ± 1.29

The zeta potential measurements revealed substantial changes in the electrokinetic properties of MON following cation modification and subsequent AHS adsorption (Table 1). Pristine MON exhibited a zeta potential of −34.3 mV, whereas MON-Na, MON-Ca, and MON-Fe exhibited values of −21.5, −8.5, and +27.0 mV, respectively. Following AHS adsorption, the zeta potentials of MON-AHS, MON-Na-AHS, MON-Ca-AHS, and MON-Fe-AHS were −37.4, −27.5, −28.7, and −30.1 mV, respectively. The shift from a positive to a negative zeta potential for MON-Fe indicates a substantial change in the electrokinetic properties of the particle-solution interface. The negative zeta potentials measured for all AHS-treated samples are consistent with an increased contribution of negatively charged organic functionalities near the slipping plane. The magnitude of the changes varied among the modified MON, potentially reflecting differences in interfacial composition, surface charge compensation, and the distribution of charged functional groups. In agreement with the TOC results, Ca-MON and Fe-MON exhibited greater carbon retention than pristine MON and Na-MON.

The measured pH values provide additional context for interpreting these changes (Table 1). The pristine AHS solution had a pH of 8.31, whereas the measured pH values of pristine MON, Na-MON, Ca-MON, and Fe-MON were 4.04, 2.89, 2.96, and 1.37, respectively. After AHS adsorption, the corresponding MON-AHS, MON-Na-AHS, MON-Ca-AHS, and MON-Fe-AHS samples exhibited pH values of 7.86, 8.03, 8.01, and 7.46, respectively. The lower pH of MON-Fe-AHS relative to the other AHS-treated samples indicates differences in the acid–base conditions of the measured systems. These differences may affect the protonation of organic functional groups and mineral surface hydroxyl groups, thereby influencing the measured zeta potential. Consequently, the observed electrokinetic changes should be interpreted in conjunction with the pH data rather than as a direct measure of carbon retention or a specific adsorption mechanism.

Overall, these results indicate that the identity of the modifying cation affects the interfacial properties and organic carbon retention of the MON-AHS systems. Nevertheless, the relative contributions of electrostatic interactions, cation-mediated associations, and interactions involving Fe-containing surface species cannot be determined unambiguously from these measurements alone. Furthermore, K^+^ introduced during the KOH-mediated synthesis of AHS may influence the ionic environment and contribute to cation redistribution during AHS-clay interactions. Accordingly, specific cation-bridging or metal–oxygen coordination mechanisms cannot be conclusively established from the available evidence.

As shown in Fig. 4a, the FTIR spectra of the AHS-treated samples remain very similar to those of pristine and cation-modified MON, indicating that the characteristic vibrational features of the aluminosilicate structure of MON are largely preserved after AHS adsorption. Only minor spectral changes were observed in the FTIR spectra. The broad band in the 3600–3200 cm^−1^ region becomes slightly more intense and broader after adsorption, potentially reflecting contributions from hydroxyl groups in AHS and adsorbed water molecules. A slight increase in the intensity of the band near 1640 cm^−1^ is also detected. This band is primarily associated with the H–O–H bending vibration of adsorbed water, although contributions from carbonyl-containing groups in AHS may overlap in this region. Minor intensity changes around 1400 cm^−1^ may be associated with symmetric stretching vibrations of carboxylate groups and are consistent with the presence of AHS-derived organic functionalities in the treated samples. Consequently, the limited spectral changes demonstrate that AHS adsorption does not substantially alter the characteristic FTIR features of MON. The carbon retention, which reached a maximum of 25.8% for MON-Fe (Table 2), may account, in part, for the predominance of the characteristic mineral bands in the FTIR spectra and the comparatively subtle spectral contributions of AHS-derived organic functionalities. Similar spectral characteristics have been reported for NHS associated with smectite minerals, where the mineral contribution remains dominant at relatively low organic loadings.^40^

**Fig. 4:**
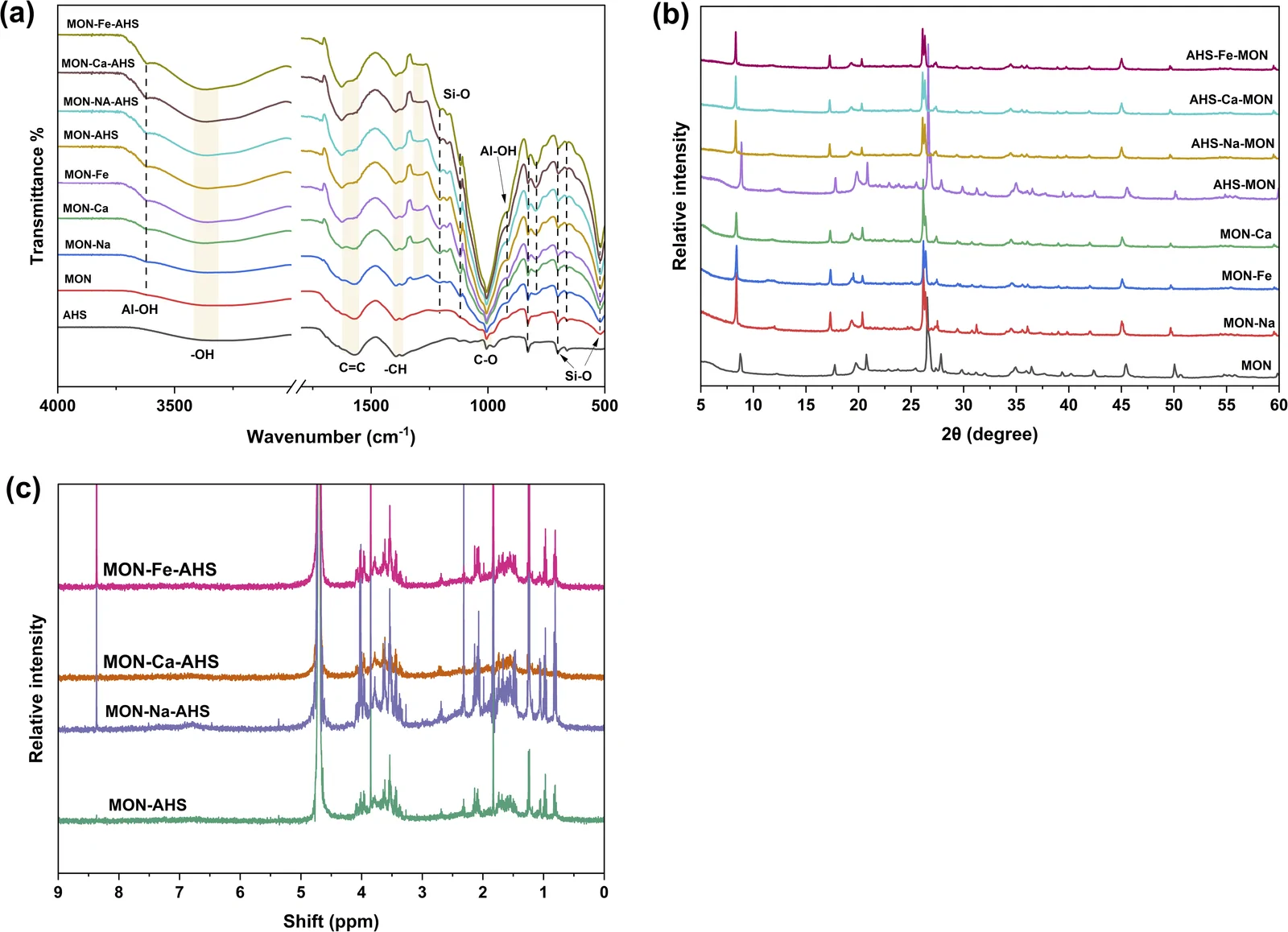
(a) FTIR spectra of AHS-MON complexes, (b) XRD spectra of pristine MON, cation-modified MON before and after AHS adsorption, and (c) ^1^H NMR spectra of the supernatant recovered after AHS adsorption.

ICP-OES analysis revealed substantial changes in the elemental composition of MON following cation modification and subsequent AHS treatment (Table S3). Cation modification resulted in pronounced enrichment of the corresponding elements in the recovered solids. Specifically, the Na, Ca, and Fe concentrations increased from 0.713 ± 0.011, 1.82 ± 0.010, and 5.61 ± 0.209 mg g^−1^ in pristine MON to 50.0 ± 1.80, 34.5 ± 0.41, and 77.8 ± 2.08 mg g^−1^ in Na-MON, Ca-MON, and Fe-MON, respectively. Following AHS treatment, the corresponding concentrations decreased to 2.20 ± 0.001 mg g^−1^ Na in AHS-Na-MON, 6.63 ± 0.061 mg g^−1^ Ca in AHS-Ca-MON, and 20.3 ± 0.46 mg g^−1^ Fe in AHS-Fe-MON. These decreases indicate that AHS treatment substantially altered the elemental composition of the recovered cation-modified clays. The reduction in the concentrations of the introduced cations coincided with pronounced K enrichment, suggesting that redistribution of cations may occur during contact with the K-rich AHS solution. In particular, K^+^ may compete with exchangeable cations for negatively charged sites within the montmorillonite structure, potentially promoting the displacement of previously introduced cations into the aqueous phase. The extent of this process may depend on the identity and initial abundance of the modifying cation, as well as its association with the mineral and organic components. However, the observed concentration changes also reflect the composition of the recovered solids and do not, by themselves, quantify the extent of cation exchange.

A pronounced increase in K concentration was observed following AHS treatment, rising from 0.812 to 46.4 mg g^−1^ in the Na-modified system, from 1.62 to 55.1 mg g^−1^ in the Ca-modified system, and from 1.03 to 66.2 mg g^−1^ in the Fe-modified system. This consistent enrichment is particularly significant because KOH was used during AHS synthesis, making the AHS solution a potential source of K^+^ during treatment. The accumulation of K in the recovered solids is consistent with the retention of K-containing species through ion exchange, association with the organic fraction, or retention of residual K-containing salts. Among these possibilities, exchange of K^+^ with previously introduced exchangeable cations provides a plausible explanation for the concurrent decrease in solid-phase Na, Ca, and Fe concentrations. However, the contribution of this process cannot be isolated from the available elemental concentrations. The pronounced K enrichment therefore highlights the importance of considering the K-rich ionic environment of the AHS solution when interpreting changes in the composition and interfacial properties of the treated clays.

The liquid-phase ICP-OES measurements provide complementary evidence of changes in elemental distribution during AHS treatment (Table S4). The Fe concentration in the liquid sample from the AHS-Fe-MON system reached 571 ± 4.89 mg L^−1^, compared with 3.44 ± 0.245 mg L^−1^ in the AHS solution. Similarly, the Na concentration in the liquid sample from the AHS-Na-MON system reached 739 ± 3.12 mg L^−1^, compared with 28.9 ± 3.19 mg L^−1^ in the AHS solution. These elevated concentrations are consistent with substantial contributions from the mineral-treatment systems to the measured aqueous elemental composition. For Na-MON, the high liquid-phase Na concentration, together with the lower Na concentration in the recovered solid after AHS treatment and the marked K enrichment, is consistent with possible K^+^-induced displacement of exchangeable Na^+^. In the Fe-modified system, the high liquid-phase Fe concentration may reflect mobilization of Fe-containing species during treatment, potentially involving dissolution or redistribution of Fe-bearing surface species. However, ICP-OES measures total elemental Fe rather than its oxidation state or chemical form; therefore, the measured concentration does not distinguish dissolved Fe ions from other Fe-containing species included in the analyzed liquid fraction. These observations support the occurrence of substantial changes in aqueous elemental composition.

Overall, the solid- and liquid-phase ICP-OES results suggested substantial cation-dependent changes in elemental composition during AHS treatment, characterized by decreases in the measured solid-phase concentrations of Na, Ca, and Fe and pronounced K enrichment. The combination of these trends is consistent with cation redistribution in the K-rich treatment environment, potentially involving competition between K^+^ and previously introduced cations for exchangeable sites. For Fe-MON, additional processes involving Fe hydrolysis, association with organic functional groups, and mobilization of Fe-containing species may also contribute to the observed changes. In conjunction with the TOC, zeta-potential, pH, and STEM-EDX results, the ICP-OES data support cation-dependent differences in the composition and interfacial behavior of the AHS-treated materials. However, the relative contributions of exchange, organic association, and Fe-containing phase formation cannot be uniquely assigned from elemental concentrations alone.

BET analysis (Table 1) indicated substantial changes in the N_2_-accessible surface area and pore volume of MON following AHS adsorption. The surface area decreased from 216.4 to 58.5 m^2^ g^−1^ for Na-MON, from 128.2 to 43.9 m^2^ g^−1^ for Ca-MON, and from 215.9 to 46.2 m^2^ g^−1^ for Fe-MON. These reductions, together with the corresponding decreases in pore volume, indicate a marked loss of accessible surface area and porosity following treatment. This behavior is consistent with the association of organic matter with the clay particles, which may reduce N_2_ accessibility through surface coverage, partial pore obstruction, or changes in particle aggregation. The extent of the decrease also varies among the cation-modified systems and should be interpreted alongside the TOC results, which indicate greater organic carbon retention by Ca-MON and Fe-MON than by Na-MON. Changes in the reported average pore width provide additional information about the textural properties of the treated materials. Na-MON-AHS and Ca-MON-AHS exhibited average pore widths of approximately 10 nm, compared with approximately 6 nm before AHS adsorption. These increases may reflect changes in the pore-size distribution or aggregate structure rather than the formation of new pores or uniform pore enlargement. In contrast, Fe-MON-AHS retained an average pore width of approximately 6 nm despite substantial decreases in specific surface area and pore volume. This finding indicates a reduction in N_2_-accessible surface area and pore volume without a comparable increase in average pore width. Overall, the BET results support substantial changes in accessible porosity of the MON following AHS, consistent with organic matter association and possible changes in pore accessibility and particle aggregation.

The XRD patterns of AHS-modified MON are shown in Fig. 4b. The characteristic diffraction reflections of MON remained evident following cation modification and AHS adsorption, indicating the preservation of the principal crystalline framework. The basal spacing *d*_001_ was calculated from the position of the (001) reflection using Bragg's law (Eqn (7)). Based on the observed peak maxima, the basal reflections of Na-MON, Fe-MON, and Ca-MON occurred at approximately 8.39°, 8.40°, and 8.38° (2*θ*), corresponding to calculated *d*_001_ values of 10.53, 10.52, and 10.54 Å, respectively. Following AHS adsorption, the basal reflection of AHS-MON was observed at approximately 8.87° (2*θ*), corresponding to a *d*_001_ value of 9.96 Å, whereas MON-Fe-AHS and MON-Ca-AHS exhibited reflections at approximately 8.33° (2*θ*), corresponding to basal spacings of approximately 10.61 Å. These basal-reflection positions suggested that the principal crystalline framework was largely preserved. Although differences in the apparent positions of the basal (001) reflections were observed among the samples, these differences alone do not establish a specific structural transformation or confirm the incorporation of AHS into the interlayer space. The XRD patterns therefore provide evidence for the retention of the characteristic crystalline features of MON following AHS adsorption, but do not conclusively establish the location of the retained organic fraction. In particular, the available XRD data do not unambiguously distinguish between association with external surfaces, incorporation into interlayer spaces, and retention in interparticle regions. Accordingly, the XRD findings should be interpreted alongside the FTIR, BET, and other characterization results, without assigning a definitive location or mechanism to the retained organic fraction.

SEM observations (Fig. S1) represent that the characteristic platelet-like morphology and aggregated structure of MON are largely preserved after cation modification and AHS adsorption. No pronounced changes are observed before and after adsorption of AHS on cation-modified MON, suggesting that these treatments do not significantly alter the overall particle morphology. These observations are consistent with the FTIR and XRD results, which collectively support the association of AHS-derived organic matter with the modified MON without clear evidence of substantial disruption of the clay structure.

STEM-EDX analysis further revealed compositional and spatial differences between Fe-MON and AHS-Fe-MON (Fig. S2). Following AHS treatment, the relative carbon content increased from 13.82 to 26.02 wt%, while K was detected at 4.04 wt%. These changes are consistent with the association of carbon-rich organic matter and K-containing species with the Fe-modified MON. The Fe elemental map of AHS-Fe-MON also revealed needle-like Fe-rich particles. Although this morphology indicates localized Fe enrichment, STEM-EDX alone does not allow the identification of the particle phase or establish whether these structures formed during AHS treatment or were already present in the starting material. In contrast, the relative Fe content decreased from 6.40 to 5.72 wt%. Because EDX provides normalized elemental compositions for the analyzed regions, this decrease does not necessarily indicate Fe loss from the bulk material and may partly reflect the increased contribution of carbon-rich material to the overall detected composition. The increased relative carbon content, detection of K, and spatially localized Fe-rich features collectively indicate compositional heterogeneity associated with AHS treatment. However, elemental mapping does not resolve Fe chemical speciation or establish a specific Fe-mediated coordination mechanism with AHS-derived functional groups.

The ^1^H NMR spectra of the liquid phase recovered after adsorption reveal cation-dependent changes in the composition of the residual dissolved organic matter (Fig. 4c and Table S2). The relative contribution of region III (3–4.3 ppm) decreases from 43.0% in the initial AHS solution to 30.1% and 23.1% after treatment with Ca-MON and Fe-MON, respectively. This decrease indicates a lower relative contribution of proton environments associated with oxygenated organic compounds in the residual solution. Accordingly, the sharp resonances in the 3.5–4.5 ppm region became considerably less intense after adsorption (Fig. 4c). These signals may arise from protons in oxygenated environments, including those associated with methoxyl groups and oxygen-bearing aliphatic structures; however, their assignment to specific functional groups or compounds requires caution because of signal overlap in the complex AHS mixture.

In contrast, the relative contribution of region I increases, especially following treatment with Ca-MON and Fe-MON. Because the reported values represent relative signal contributions, this increase does not necessarily indicate an absolute enrichment of aliphatic compounds in the residual solution. The resonance at approximately 2.30 ppm was substantially reduced and remains weakly detectable after adsorption onto Ca-MON.

Overall, the NMR results indicate that AHS treatment alters the relative composition of the residual dissolved organic matter, with more pronounced changes in regions associated with oxygenated organic environments for Ca-MON and Fe-MON. These observations are consistent with the TOC results showing greater organic carbon retention by the Ca- and Fe-modified clays. Together with the zeta-potential measurements, they support cation-dependent interactions between the dissolved organic fraction and the modified mineral surfaces.

Fig. 5a shows a marked enrichment of carbon in the near-surface regions of all three cation-modified clays following AHS treatment, as determined by XPS survey quantification (Fig. S5 and Table S7). To express this enrichment relative to the aluminosilicate substrate, the carbon concentration was normalized to the principal framework elements using the C/(Si + Al) atomic ratio. This ratio increased from 0.089 to 0.792 for the Na system, from 0.127 to 0.266 for the Ca system, and from 0.144 to 0.534 for the Fe system, giving the order Na > Fe > Ca for the final near-surface ratios. The increase in C/(Si + Al), together with the attenuation of the Si and Al signals, is consistent with the accumulation of an AHS-rich phase at the external surfaces of the clay particles. Since conventional XPS probes only the outermost few nanometers, these values represent the near-surface composition and should not be taken as a direct measure of the total AHS content of the bulk composites. Consistently, only minor changes were observed in the FTIR and XRD patterns following AHS adsorption, while the characteristic vibrational bands and diffraction reflections of MON were largely preserved. However, the available XPS, XRD, and BET measurements do not uniquely determine the spatial distribution of the retained organic fraction. In particular, the absence of a clearly resolved shift in the basal (001) reflection provides no direct evidence of basal expansion following AHS treatment. External-surface association and retention in interparticle regions are plausible, while interlayer incorporation cannot be established from the present data.

**Fig. 5:**
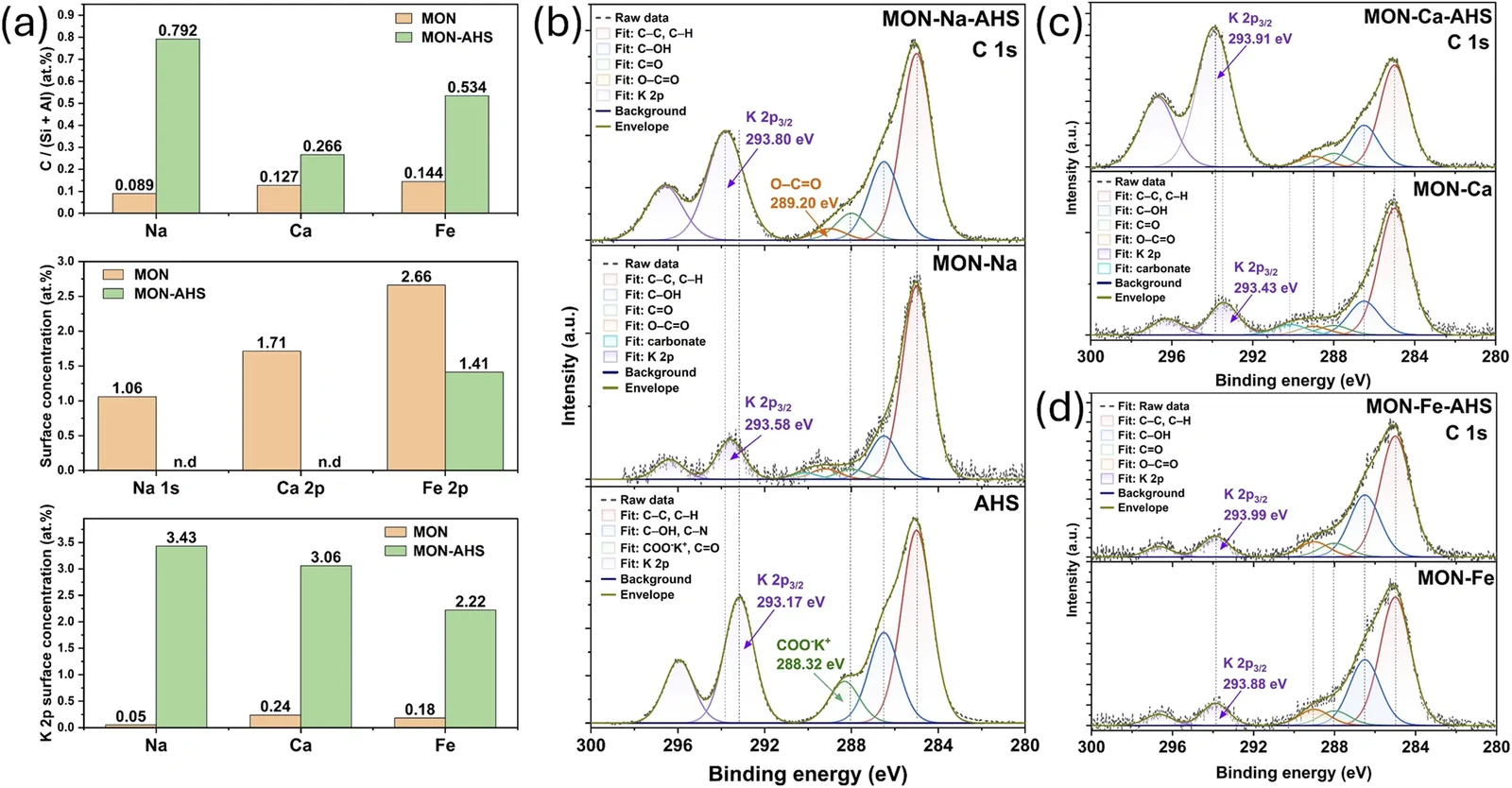
XPS analysis of cation-modified MON before and after AHS adsorption. (a) Survey-derived C/(Si + Al) atomic ratios and surface concentrations of Na, Ca, Fe, and K. High-resolution C 1s K^−1^ 2p spectra and curve fits of the (b) Na, (c) Ca, and (d) Fe systems; the spectrum of isolated AHS is included for comparison.

Fig. 5b–d and Table S7 show pronounced K enrichment in the near-surface regions following AHS treatment. The measured K content increased from 0.05 to 3.43 at% in the Na system, from 0.24 to 3.06 at% in the Ca system, and from 0.18 to 2.22 at% in the Fe system. This increase is attributed, at least in part, to the contribution of K-containing species from the KOH-based AHS solution^71,72^ (Fig. 5 and S5). In contrast, the Na signal decreased from 1.06 at% to below the survey-XPS quantification limit, while the Ca signal decreased from 1.71 at% to below the corresponding limit. The surface Fe concentration also decreased, from 2.66 to 1.41 at%, although Fe remained detectable after AHS treatment. These changes are consistent with a modification of the relative cation contributions within the near-surface region following AHS adsorption. In the Na- and Ca-derived composites, the K signal became the predominant metal-ion contribution detected by XPS, which may largely reflect the accumulation of K-containing AHS species at the clay surface. In the Fe-derived composite, both Fe and K remained evident in the near-surface composition, suggesting the coexistence of Fe-containing clay sites and adsorbed K-containing AHS species. Such behavior may primarily reflect the accumulation of K-containing AHS species at the clay surface; however, XPS survey quantification alone cannot distinguish these contributions or establish the extent of cation exchange.

The high-resolution K 2p spectra provide additional information on the chemical environment of surface-detected potassium. The K 2p_3/2_ signal of the isolated AHS was observed at 293.17 eV, whereas a minor K contribution in the reference clay was present at 293.52 eV (Fig. S3). Following AHS adsorption, the surface concentration of K increased markedly in all composites, and the K 2p_3/2_ component was located at 293.80, 293.91, and 293.99 eV for AHS-Na-MON, AHS-Ca-MON, and AHS-Fe-MON, respectively. The positive shift in the K 2p_3/2_ binding energy by approximately 0.63–0.82 eV relative to that of the isolated AHS is consistent with a change in the average local coordination environment of K^+^ during composite formation. A proportion of the K^+^ may remain associated with the deprotonated oxygen-containing groups of AHS, whereas another proportion of K in the reference clay may be associated with mineral surface or accessible interlayer environments. Following composite formation, K may experience a different distribution of local electrostatic and coordination environments involving AHS-derived and mineral-derived oxygen atoms. Previous XPS studies of phyllosilicates have reported that variation in K 2p binding energy may associate with changes in the local environment of potassium and its interactions with mineral oxygen atoms.^73,74^ Nevertheless, the observed binding-energy shifts do not uniquely identify the coordinating oxygen atoms or establish a specific K–O coordination geometry. Because binding-energy assignments can also be affected by charge referencing, differential charging, and spectral-fitting procedures, the shifts should be interpreted as evidence of a change in the measured chemical environment rather than definitive proof of K-mediated interfacial coordination.

The differences between the XPS and TOC results further highlight the distinction between near-surface composition and bulk carbon retention. TOC analysis showed that carbon retention increased in the order Na-MON < Ca-MON < Fe-MON, with values of 316, 413, and 546 mg C per g clay, respectively. In contrast, the final XPS-derived C/(Si + Al) atomic ratios followed the order Na > Fe > Ca. These trends are not necessarily inconsistent because TOC quantifies retained carbon throughout the recovered solid, whereas XPS characterizes the outermost near-surface region. The difference may reflect variations in the relative distribution of carbon-rich material between the analyzed surface region and the bulk composite, as well as differences in the attenuation of the underlying Si and Al signals. The results suggest that the identity of the modifying cation influences both total organic carbon retention and the resulting near-surface composition, although these measurements do not provide sufficient spatially resolved information to establish the distribution of AHS within the composites.

The Fe-modified MON exhibited the highest bulk carbon uptake according to TOC analysis (Table 2), while a distinct Fe contribution remained within the near-surface region following AHS treatment (Fig. 5a). Analysis of the Fe 2p_3/2_ spectra using constrained multiplet fitting yielded an increase in the relative contribution assigned to Fe^2+^ from 9.9% in MON-Fe to 19.3% in MON-Fe-AHS, accompanied by a decrease in the fitted Fe^3+^/Fe^2+^ area ratio from 9.11 to 4.18. The components assigned to Fe^3+^ nevertheless accounted for the greater proportion of the fitted main-multiplet area in both samples (Fig. S4, Table S5 and S6). This difference may reflect changes in the near-surface Fe chemical environment following AHS adsorption, including possible variations in local coordination or oxidation-state distribution. However, owing to the overlap of the Fe^2+^ and Fe^3+^ multiplet structures and the uncertainties associated with the constrained fitting model, the observed variation cannot be assigned unambiguously to Fe(iii) reduction. The derived proportions should therefore be regarded as model-dependent estimates of the relative Fe spectral contributions.

Overall, the XPS results indicate pronounced enrichment of carbon and potassium within the near-surface regions of the clay particles following AHS treatment, accompanied by changes in the relative contributions of the detected cations. These observations are consistent with the accumulation of K-containing AHS species at the clay surface and attenuation of signals from the underlying mineral phase. A contribution from the redistribution of cations among accessible surface or interlayer environments cannot be excluded. In the Fe-containing system, the variation in the fitted Fe^2+^ contribution may further reflect changes in the near-surface Fe chemical environment, although the present evidence does not establish the occurrence of an interfacial redox process. The combined XPS, TOC, zeta-potential, FTIR, and adsorption results are therefore consistent with possible cation-dependent interactions contributing to AHS retention, without establishing a specific interfacial bonding mechanism.

### 3.5. Adsorption and desorption experiments

The adsorption of AHS onto Na-MON, Ca-MON, and Fe-MON was evaluated using the Langmuir, Freundlich, and Sips isotherm models (2–4). Fig. 6 presents the experimental adsorption data and the corresponding model fits, while the obtained isotherm parameters from the fitting are summarized in Table 3.

**Fig. 6:**
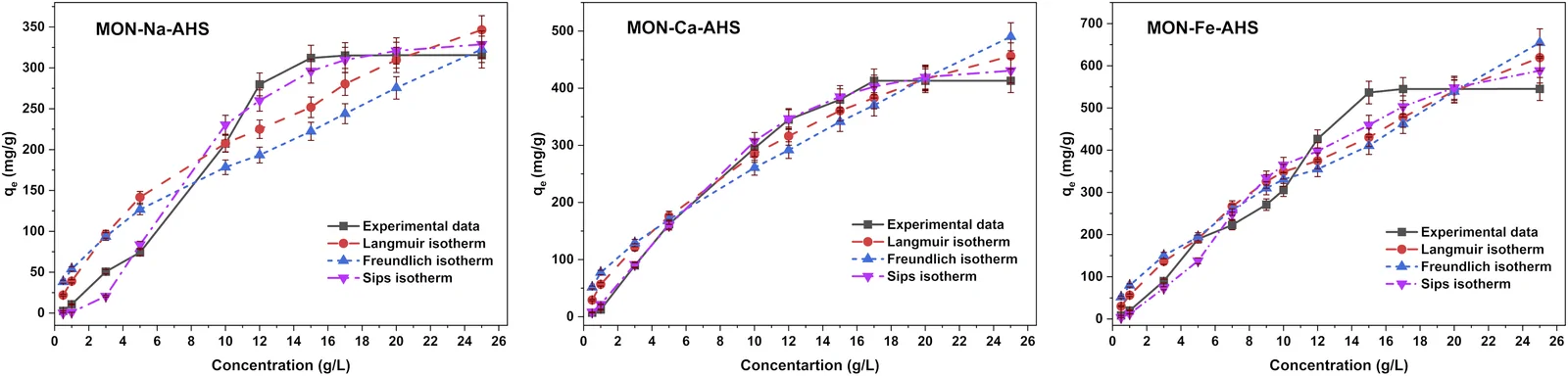
Adsorption isotherms of AHS on Na-, Ca-, and Fe-modified MON.

**Table 3:** Fitting parameters of isotherm models for the adsorption of AHS by Na-MON, Ca-MON, and Fe-MON

Sample	Isotherm model	Parameter	Estimate	95% confidence interval	*R* ^2^	RMSE	AICc
MON-Na-AHS	Langmuir	*q*_max_ (mg C per g clay)	460.8	403.66–491.83	0.927	40.824	79.90
MON-Na-AHS	Langmuir	*K*_L_ (L per mg per C)	0.105	0.07466–0.14433			
MON-Na-AHS	Freundlich	*K*_F_ (mg C per g clay)(L per mg per C)^1/*n*_F_^	61.2	7.15–90.65	0.875	55.500	86.04
MON-Na-AHS	Freundlich	*n*_F_	1.91	0.779–2.220			
MON-Na-AHS	Sips	*q*_max_ (mg C per g clay)	335.7	294.76–376.67	0.985	15.824	65.23
MON-Na-AHS	Sips	*K*_s_ ((L per mg per C)^1/*n*_s_^)	0.163	0.13463–0.19186			
MON-Na-AHS	Sips	*n*_s_	3.03	1.783–4.285			
MON-Ca-AHS	Langmuir	*q*_max_ (mg C per g clay)	659.8	467.57–852.22	0.969	27.825	72.23
MON-Ca-AHS	Langmuir	*K*_L_ (L per mg per C)	0.107	0.0377–0.1773			
MON-Ca-AHS	Freundlich	*K*_F_ (mg C per g clay)(L per mg per C)^1/*n*_F_^	83.8	35.75–131.81	0.917	45.898	82.24
MON-Ca-AHS	Freundlich	*n*_F_	1.72	1.063–2.377			
MON-Ca-AHS	Sips	*q*_max_ (mg C per g clay)	445.8	415.70–489.04	0.982	8.52	52.84
MON-Ca-AHS	Sips	*K*_s_ ((L per mg per C)^1/ns^)	0.215	0.1838–0.2463			
MON-Ca-AHS	Sips	*n*_s_	2.41	1.500–2.623			
MON-Fe-AHS	Langmuir	*q*_max_ (mg C per g clay)	1080.2	808.9–1251.5	0.927	53.820	100.99
MON-Fe-AHS	Langmuir	*K*_L_ (L per mg per C)	0.068	0.0059–0.1315			
MON-Fe-AHS	Freundlich	*K*_F_ (mg C per g clay)(L per mg per C)^1/*n*_F_^	92.13	39.26–144.99	0.890	66.031	105.90
MON-Fe-AHS	Freundlich	*n*_F_	1.52	0.980–2.052			
MON-Fe-AHS	Sips	*q*_max_ (mg C per g clay)	650.5	450.7–850.3	0.955	42.342	98.90
MON-Fe-AHS	Sips	*K*_s_ ((L per mg per C)^1/*n*_s_^)	0.163	0.0965–0.2303			
MON-Fe-AHS	Sips	*n*_s_	1.94	0.811–2.061			

The performance of the Langmuir, Freundlich, and Sips isotherm models was evaluated using the coefficient of determination (*R*^2^), root-mean-square error (RMSE), and corrected Akaike information criterion (AICc), considering both the agreement between predicted and experimental uptake and the complexity of the fitted models. For Na-MON and Ca-MON, the Sips model consistently provided the strongest statistical support, exhibiting the highest *R*^2^ values (0.985 and 0.982, respectively), the lowest RMSE values (15.824 and 8.52 mg C per g clay, respectively), and substantially lower AICc values than either the Langmuir or Freundlich model. For Fe-MON, the Sips model likewise yielded the lowest RMSE (42.342 mg C per g clay) and AICc (98.90); however, the difference between the Sips and Langmuir AICc values was only 2.09, indicating that the evidence favoring Sips over Langmuir was less decisive for this system. Overall, the model-comparison results favor the Sips model, particularly for Na-MON and Ca-MON, while indicating a smaller improvement over the Langmuir model for Fe-MON.

Overall, the Sips model provided the best statistical fit to the experimental adsorption data among the three models evaluated, although the magnitude of its improvement varied across the modified MON systems. However, the superior fit does not, by itself, establish surface heterogeneity or the involvement of multiple adsorption mechanisms. The differences in AHS adsorption among Na-MON, Ca-MON, and Fe-MON may be associated with variations in surface charge, surface functional groups, and the chemical environments of the incorporated cations. Nevertheless, the isotherm analysis alone cannot identify specific adsorption sites or determine the relative contributions of electrostatic interactions, charge screening, and cation-mediated interactions. These factors should therefore be considered potential contributors to the observed adsorption behavior rather than mechanisms directly established by the present results.

The adsorption kinetics of AHS onto Na-, Ca-, and Fe-modified MON were investigated using PFO and PSO kinetic models (Fig. 7). All three systems exhibited a rapid initial increase in adsorption capacity, followed by a gradual decrease in the adsorption rate. The experimental adsorption capacities approached stable values by 24 h, with negligible changes between 24 and 48 h, indicating that an apparent adsorption plateau was reached within this period. This trend demonstrates a clear dependence of AHS adsorption on the modification of MON by cations, with the highest adsorption capacity observed for Fe-modified MON, followed by Ca- and Na-modified MON.

**Fig. 7:**
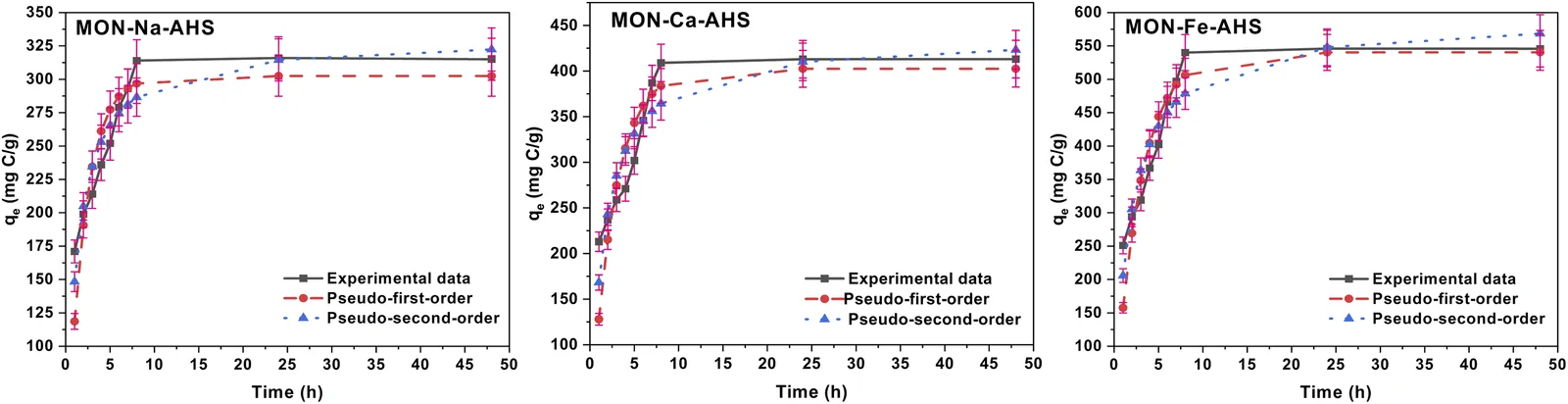
Adsorption kinetics of AHS on Na-modified, Ca-modified, and Fe-modified MON.

The performance of the PFO and PSO models was assessed using *R*^2^, RMSE, and AICc (Table 4). For Na-MON-AHS, the PSO model more closely reproduced the experimental adsorption kinetics than the PFO model, as reflected by the lower RMSE (15.497 mg C per g clay) and AICc (60.53), together with the higher *R*^2^ (0.904). A similar trend was observed for Ca-MON-AHS, for which the PSO model yielded an *R*^2^ of 0.848, an RMSE of 28.916 mg C per g clay, and an AICc of 73.00, compared with 0.765, 35.888 mg C per g clay, and 77.32, respectively, for the PFO model. Similarly, for MON-Fe-AHS, the PSO model showed a higher *R*^2^ (0.895) and lower RMSE (34.188 mg C per g clay) and AICc (76.35). Overall, the statistical comparisons favored the PSO model across all three systems, although the strength of this preference varied with the modified mineral.

**Table 4:** Kinetic parameters obtained from PFO and PSO models for AHS adsorption onto modified MON

Sample	Kinetic model	Parameter	Estimate	95% confidence interval	*R* ^2^	RMSE	AICc
MON-Na-AHS	Pseudo-first-order	*q*_e_ (mg C per g clay)	302.451	272.41–332.49	0.791	22.836	68.28
MON-Na-AHS	Pseudo-first-order	*k*_1_ (h^−1^)	0.498	0.305–0.691			
MON-Na-AHS	Pseudo-second-order	*K*_2_ (g per mg per C h)	0.00246	0.00131–0.00362	0.904	15.497	60.53
MON-Na-AHS	Pseudo-second-order	*q*_e_ (mg C per g clay)	330.599	303.35–357.85			
MON-Ca-AHS	Pseudo-first-order	*q*_e_ (mg C per g clay)	402.560	349.02–456.10	0.765	35.888	77.32
MON-Ca-AHS	Pseudo-first-order	*K*_1_ (h^−1^)	0.382	0.215–0.550			
MON-Ca-AHS	Pseudo-second-order	*K*_2_ (g per mg per C h)	0.00143	0.00051–0.00235	0.848	28.916	73.00
MON-Ca-AHS	Pseudo-second-order	*q*_e_ (mg C per g clay)	437.465	382.56–492.37			
MON-Fe-AHS	Pseudo-first-order	*q*_e_ (mg C per g clay)	540.374	480.79–599.96	0.869	38.244	78.60
MON-Fe-AHS	Pseudo-first-order	*K*_1_ (h^−1^)	0.345	0.227–0.463			
MON-Fe-AHS	Pseudo-second-order	*K*_2_ (g per mg per C h)	0.000906	0.000403–0.001409	0.895	34.188	76.35
MON-Fe-AHS	Pseudo-second-order	*q*_e_ (mg C per g clay)	590.634	522.56–658.71			

The higher adsorption capacities of Ca-MON and Fe-MON indicate that AHS uptake is influenced by the identity of the exchangeable cation. This behavior may reflect differences in surface charge and interactions between mineral-associated cations and oxygen-containing functional groups in AHS. However, the PSO model's superior fit does not establish a specific adsorption mechanism.

To assess the reversibility of AHS retention by cation-modified MON, successive adsorption–desorption experiments were conducted over four cycles (Fig. 8 and Table S9). All three systems exhibited a progressive decrease in adsorption capacity, from 316 to 109 mg C per g clay for Na-MON-AHS, from 413 to 189 mg C per g clay for Ca-MON-AHS, and from 546 to 225 mg C per g clay for Fe-MON-AHS. These changes indicate that the net removal of organic carbon from the aqueous phase decreased during repeated cycling.

**Fig. 8:**
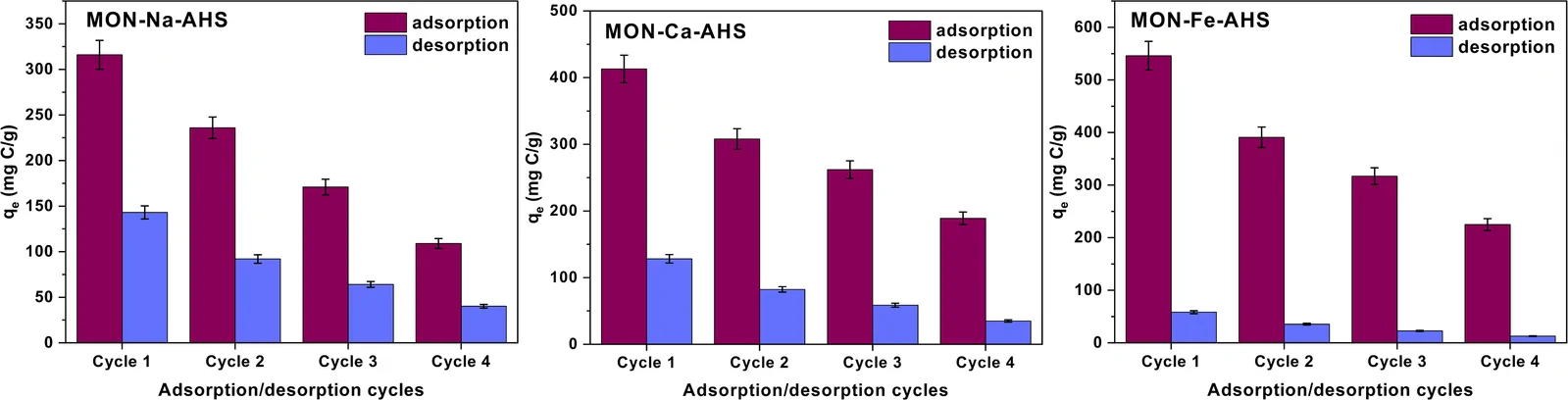
Adsorption and desorption capacities (*q*_e_) of AHS on Na-MON, Ca-MON, and Fe-MON over four consecutive adsorption–desorption cycles.

The desorption capacities (*q*_des_) also decreased progressively over the four cycles. For Na-MON-AHS, q_des_ declined from 143.15 to 40.17 mg C per g clay, while Ca-MON-AHS decreased from 128.24 to 34.97 mg C per g clay. Fe-MON-AHS exhibited the lowest desorption capacity throughout the experiment, decreasing from 58.37 to 12.83 mg C per g clay. These results indicate cation-dependent differences in the release of AHS-derived organic carbon, with Fe-MON showing comparatively lower carbon release under the investigated conditions. However, because the desorption capacities represent absolute amounts rather than fractions of the previously adsorbed carbon, they do not alone establish the relative reversibility of AHS retention. Moreover, residual organic carbon retained after each cycle may contribute to the observed changes in subsequent adsorption and desorption capacities.

Overall, the repeated-cycle experiments demonstrate differences in the uptake and release of AHS-derived organic carbon among the three cation-modified MON systems. Fe-MON exhibited the highest initial adsorption capacity and the lowest absolute desorption capacity, indicating comparatively greater carbon retention under the experimental conditions. These observations reveal differences in retention behavior but do not, by themselves, establish the molecular interactions responsible.

## 4. Conclusions

This study demonstrates that AHS produced *via* hydrothermal humification of biomass (pear pomace and pine bark) can be effectively adsorbed and retained by MON, providing a basis for investigating the incorporation of biomass-derived organic carbon into organo-mineral association. Modification of MON with Na^+^, Ca^2+^, and Fe^3+^ resulted in distinct AHS adsorption behavior, demonstrating the influence of modifying-cation identity on AHS-mineral interactions. Among the investigated materials, Fe-MON exhibited the highest adsorption capacity (546 mg C g^−1^ of MON), followed by Ca-MON and Na-MON. The differences in adsorption, together with the observed changes in surface properties and AHS composition, demonstrate that cation modification influences the interfacial characteristics of the AHS-MON systems. Electrostatic interactions and possible cation-mediated associations may contribute to AHS retention, although their individual contributions and the possible involvement of specific metal–organic interactions remain unresolved.

Structural and textural characterization indicated that the main properties of MON were largely preserved following cation modification and AHS adsorption. Nevertheless, the decreases in specific surface area and pore volume following AHS treatment indicate reduced N_2_-accessible surface area and pore accessibility. The XRD, FTIR, XPS, and STEM-EDX results provide complementary information on structural features, functional-group signatures, near-surface composition, and local elemental distribution, respectively. However, these findings do not conclusively establish specific interfacial bonding mechanisms.

The adsorption isotherms were best described by the Sips model, while the kinetic data were better represented by the pseudo-second-order model than by the pseudo-first-order model. Successive adsorption–desorption experiments further revealed cation-dependent differences in carbon retention and release. Fe-MON exhibited the highest initial adsorption capacity and the lowest absolute desorption capacity, indicating comparatively lower carbon release under the conditions tested. However, these results do not establish long-term carbon stabilization or intrinsic resistance to desorption independently of the amount of carbon retained.

Overall, this study provides a basis for further evaluating biomass-derived AHS-mineral composites for soil-related applications and carbon management. Future research should assess the persistence of mineral-associated carbon over environmentally relevant timescales, evaluate the effects of these composites on soil properties and ecological processes, and establish the environmental and process sustainability of their production and application.

## Author contributions

Khatereh Pakzad: writing – review & editing, writing – original draft, visualization, software, investigation, formal analysis, methodology, data curation, conceptualization. Giulia Ischia: writing – review & editing, visualization, formal analysis, methodology, software, data curation. Markus Antonietti: review& editing, validation, supervision, investigation, resources. Jiyong Kim: writing – review & editing, methodology, software, formal analysis. Vera Susanne Rotter: review& editing, validation, supervision, investigation. Alexander Volikov: review& editing, visualization, investigation.

## Conflicts of interest

The authors declare that they have no known competing financial interests or personal relationships that could have appeared to influence the work reported in this paper.

## Supplementary Material

RA-OLF-D6RA09407D-s001

## Data Availability

The data supporting this article have been included as part of the manuscript and its supplementary information (SI). Supplementary information: additional experimental details, supporting characterization data, supplementary figures, and tables providing further information on the synthesis, characterization, and interactions of biomass-derived artificial humic substances with clay minerals. These materials support the findings presented in the main article. See DOI: https://doi.org/10.1039/d6ra09407d.
