# Supplementary material for: Cation modification of montmorillonite regulates the retention of biomass-derived artificial humic substances
Source: RSC Adv. 2026 Nov 4. Online ahead of print. doi: 10.1039/d6ra09407d (PMC13732911; doi:10.1039/d6ra09407d)
Supplement: Supplementary file 1 [file RA-OLF-D6RA09407D-s001.pdf]

Supplementary Information (SI) for RSC Advances.
This journal is © The Royal Society of Chemistry

Supplementary Material

Cation Modification of Montmorillonite Regulates the Retention of Biomass-Derived Artificial Humic Substances

Khatereh Pakzad[a], Giulia Ischia[a], Markus Antonietti[a], Jiyong Kim[b], Vera Susanne Rotter[c], Alexander Volikov[a]

[a]Department of Colloid Chemistry, Max Planck Institute of Colloids and Interfaces, Am Mühlenberg 1, 14476 Potsdam, Germany

[b] Functional Materials and Devices, Fraunhofer Institute for Applied Polymer Research, Potsdam (IAP) 14476 Potsdam, Germany

[c]Department of Environmental Technology, Chair of Circular Economy and Recycling Technology, Technische Universität Berlin 10623, Berlin, Germany

*Corresponding author. E-Mail address: Khatereh.Pakzad@mpikg.mpg.de

Table S1. Elemental analysis of synthesized AHS.

Sample	C%	O%	H%	N%	S%	Ash%
AHS	41.6	50.2	5.3	0.8	0.9	1.2

Table S2. Relative distribution (%) of signal area over chemical shift regions (in ppm) of ^{1}H-NMR spectra of liquid phase recovered after adsorption of AHS.

Sample	I<br>0-2 ppm	II<br>2-3 ppm	III<br>3-4.3 ppm	IV<br>5-7.5 ppm
AHS	47.1	8.2	43.0	1.7
MON-AHS	44.3	4.7	50.8	0.2

MON-Na-AHS	47.8	4.8	47.0	0.4
MON-Ca-AHS	63.2	6.7	30.1	0.0
MON-Fe-AHS	66.0	4.2	23.1	6.7

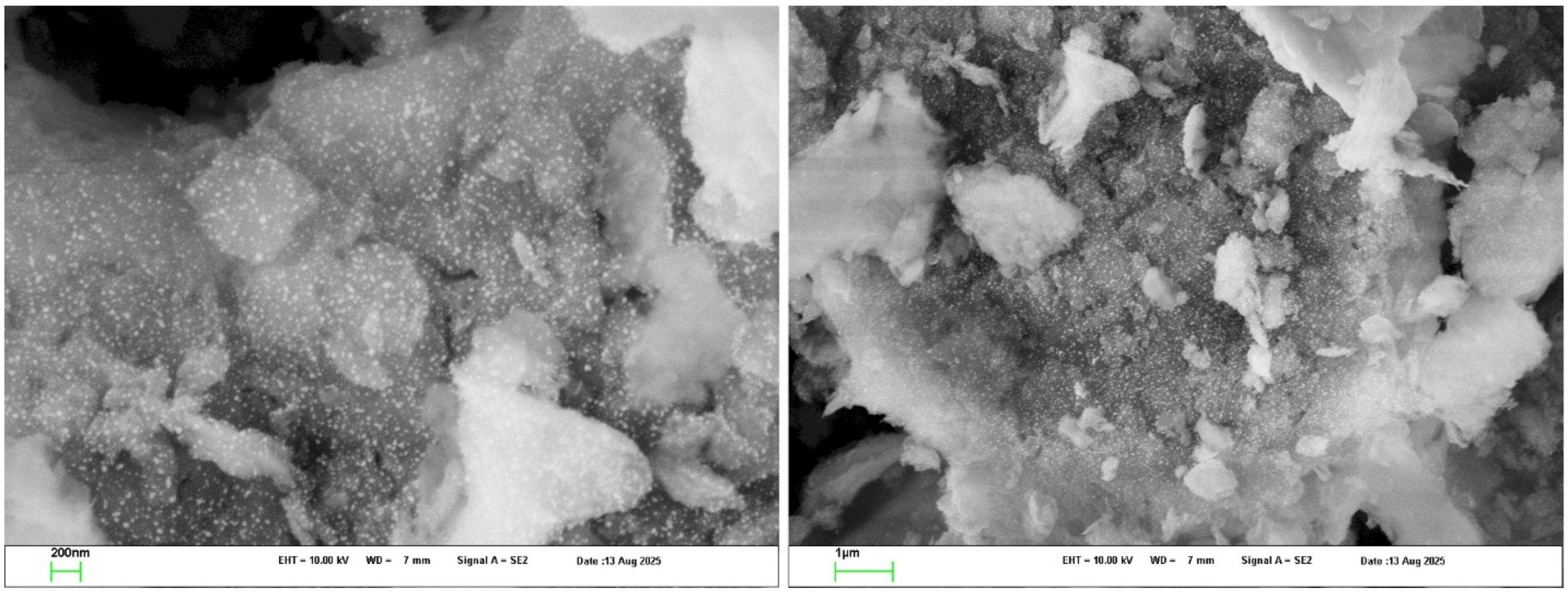

Na-montmorillonite

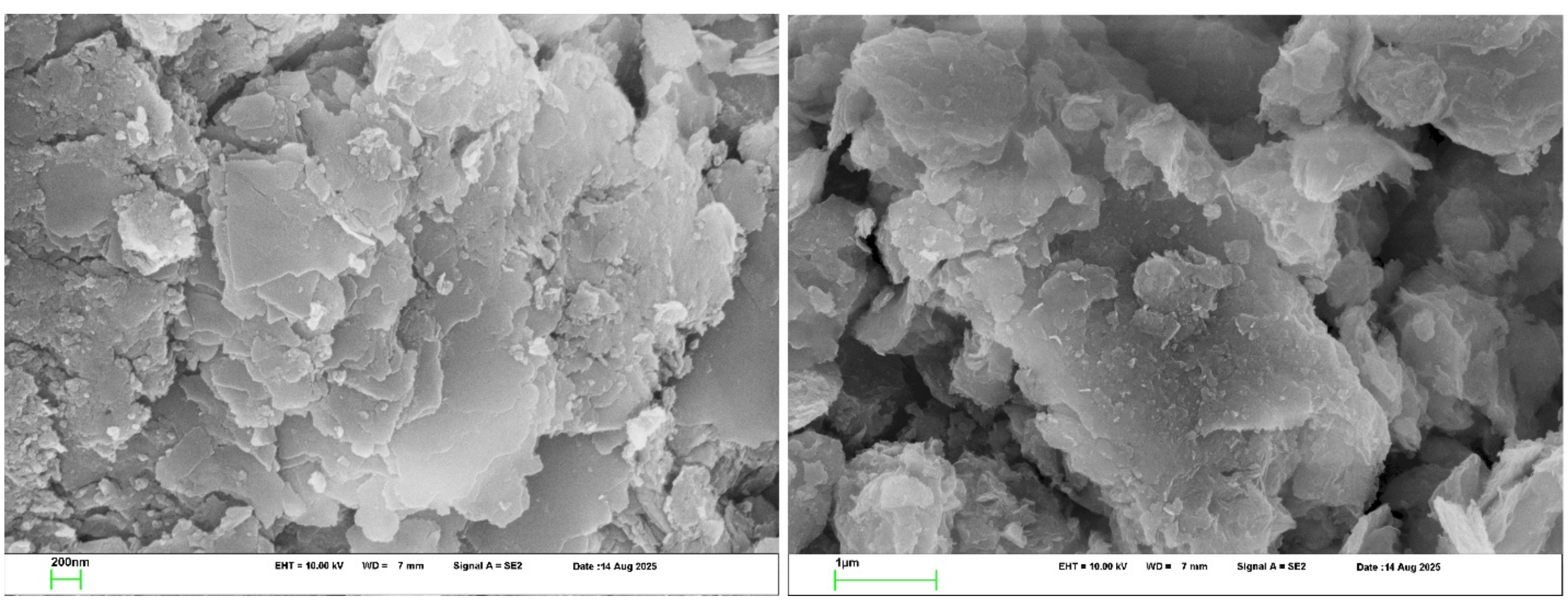

Na-montmorillonite-AHS

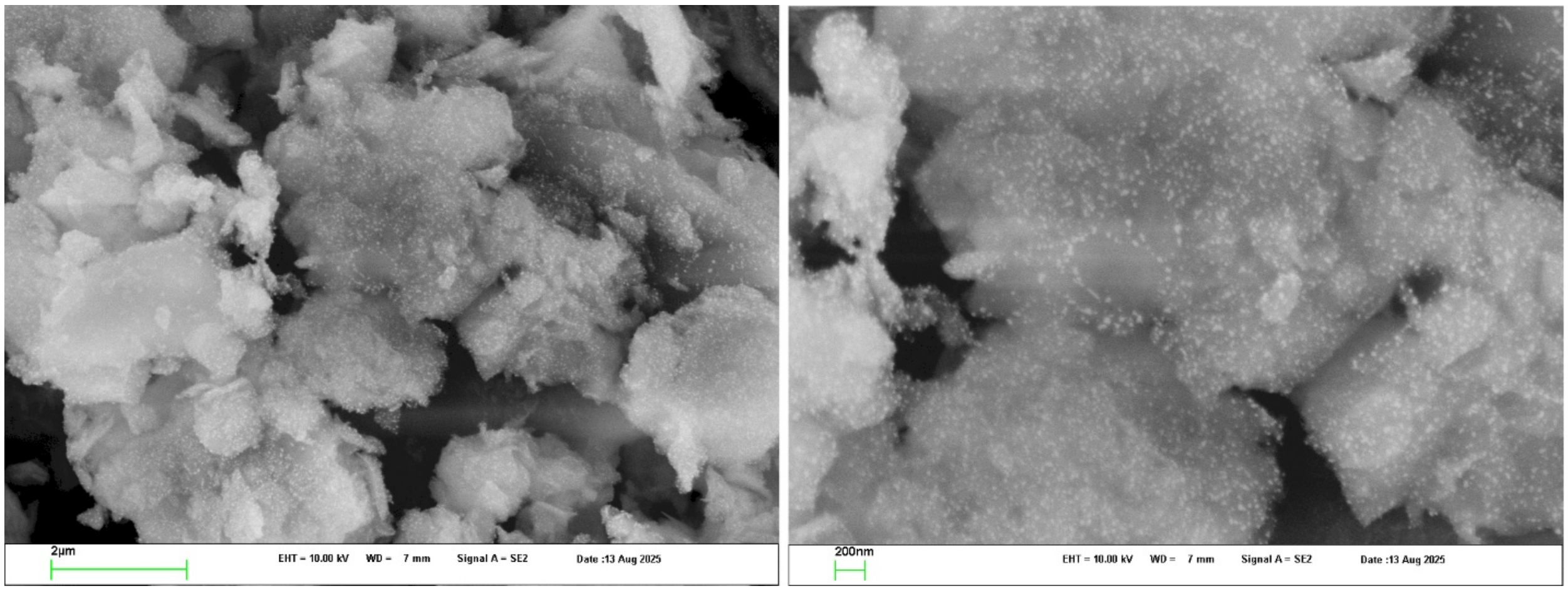

Ca-montmorillonite

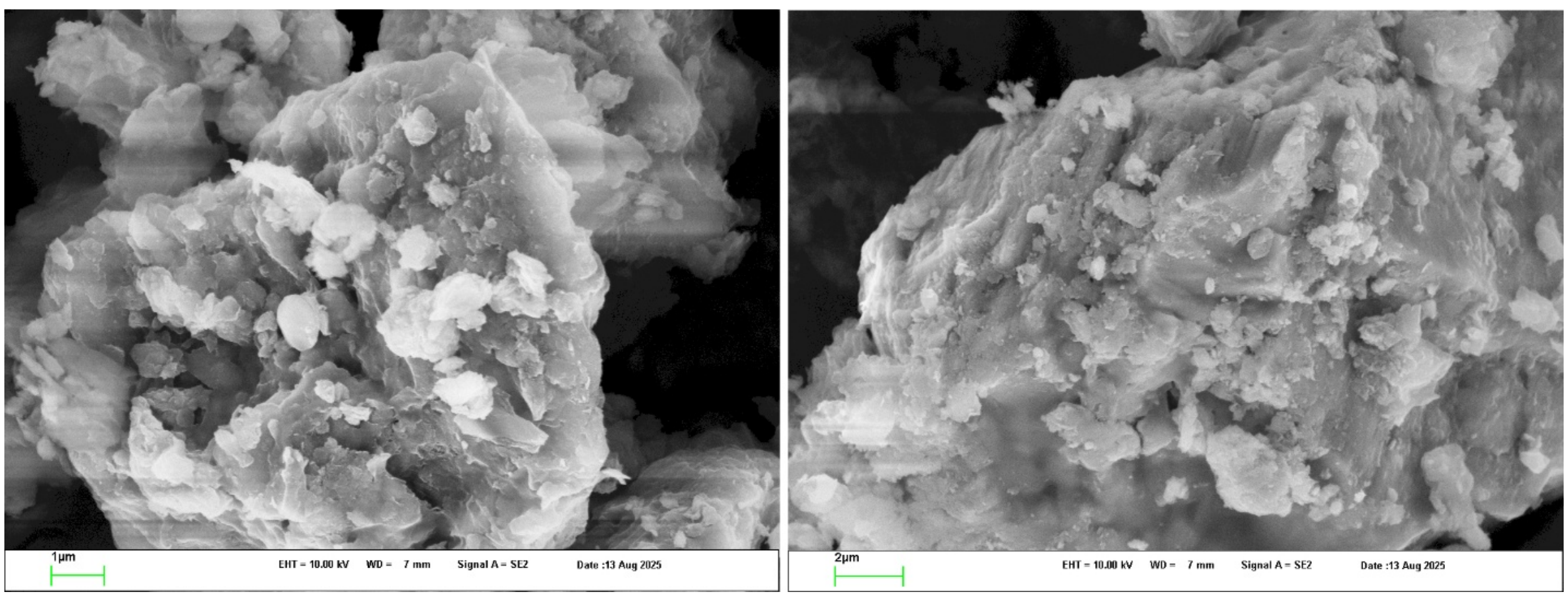

Ca-montmorillonite-AHS

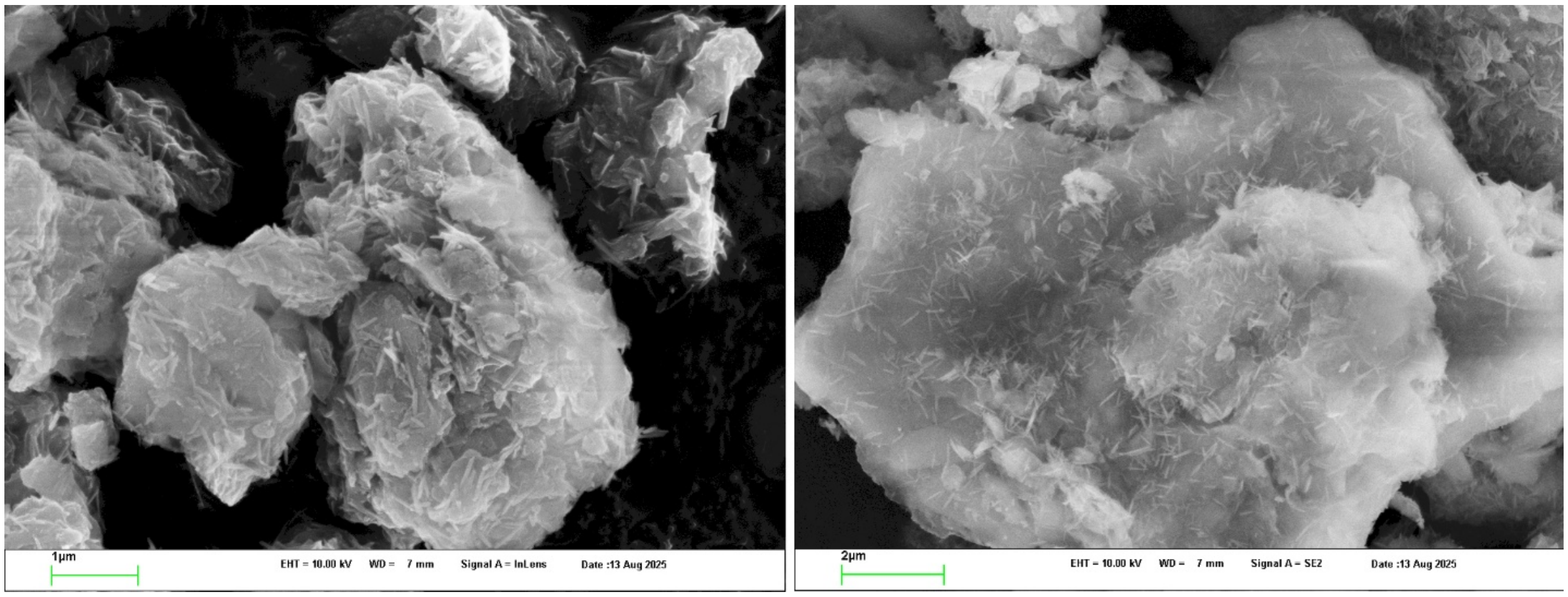

Fe-montmorillonite

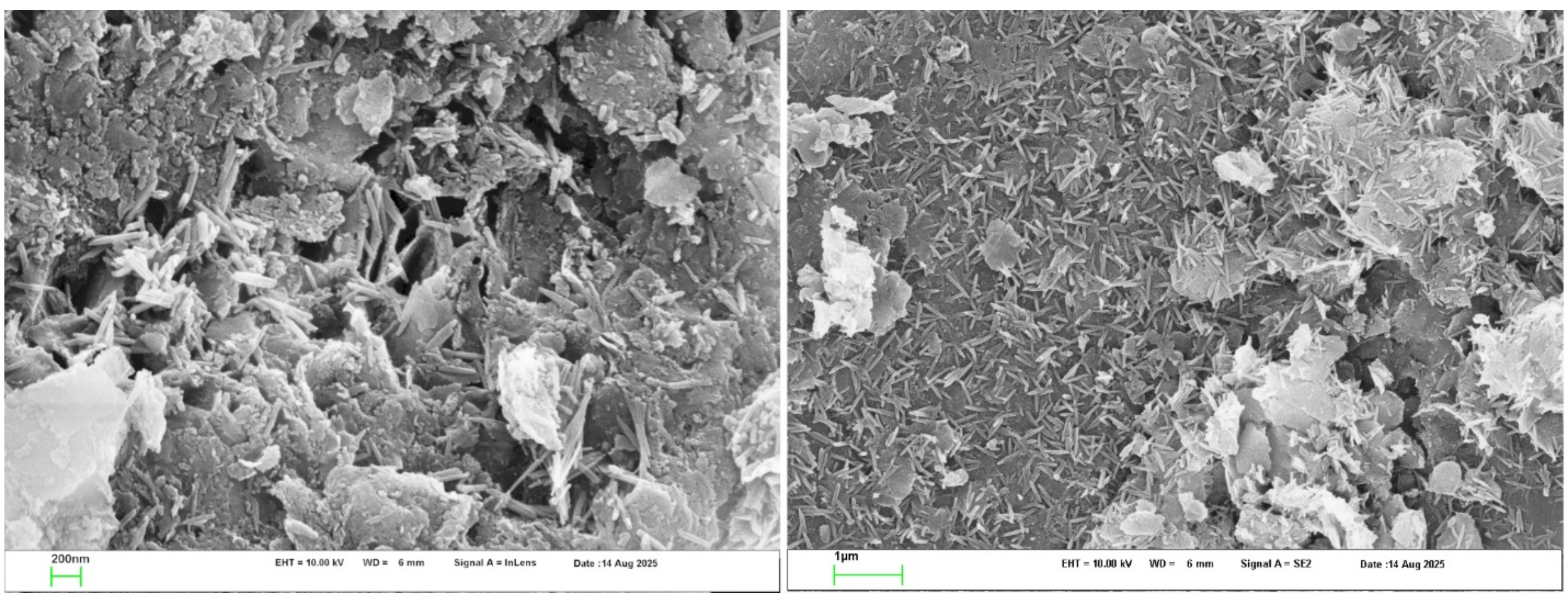

Fe-montmorillonite-AHS

Figure S1. Scanning electron microscopy images of MON in cation-modified and AHS-adsorbed forms.

Table S3. ICP–OES elemental composition (mg/g) of MON samples before and after AHS treatment. Values are presented as mean ± standard deviation.

Element	MON	AHS-MON	Na-MON	AHS-Na-MON	Ca-MON	AHS-Ca-MON	Fe-MON	AHS-Fe-MON
Na	0.713 ± 0.011	0.971 ± 0.024	50.0 ± 1.80	2.20 ± 0.001	1.69 ± 0.042	1.98 ± 0.012	1.05 ± 0.014	1.88 ± 0.013

Ca	1.82 ± 0.010	0.408 ± 0.002	0.369 ± 0.006	0.675 ± 0.003	34.5 ± 0.41	6.63 ± 0.061	0.531 ± 0.012	0.771 ± 0.008
Mg	1.87 ± 0.021	0.968 ± 0.005	1.17 ± 0.011	1.15 ± 0.026	1.08 ± 0.012	1.31 ± 0.023	1.23 ± 0.002	1.18 ± 0.007
Fe	5.61 ± 0.209	3.79 ± 0.127	4.67 ± 0.127	3.87 ± 0.076	4.21 ± 0.074	4.54 ± 0.049	77.8 ± 2.08	20.3 ± 0.46
K	0.772 ± 0.016	44.6 ± 1.02	0.812 ± 0.001	46.4 ± 0.30	1.62 ± 0.013	55.1 ± 0.53	1.03 ± 0.011	66.2 ± 3.00
Al	10.1 ± 0.11	6.07 ± 0.102	8.20 ± 0.169	6.60 ± 0.029	6.84 ± 0.117	7.13 ± 0.089	6.96 ± 0.175	5.94 ± 0.071
Mn	0.018 ± 0.0003	0.049 ± 0.0007	0.011 ± 0.0002	0.066 ± 0.0005	0.014 ± 0.0004	0.085 ± 0.0017	0.186 ± 0.0011	0.068 ± 0.0006

Table S4. Elemental concentrations in the AHS solution and liquid fractions recovered after contact of AHS with cation-modified MONs, determined by ICP-OES. Values are reported as mean ± standard deviation (mg/L).

Element	AHS Solution	AHS-Na-MON	AHS-Ca-MON	AHS-Fe-MON
Na	28.9 ± 3.19	739 ± 3.12	28 ± 1.53	27 ± 14.71
Ca	20.7 ± 0.47	22.1 ± 0.24	300 ± 8.06	19.7 ± 0.13
Mg	29.2 ± 0.12	32.7 ± 0.35	32.4 ± 0.53	31.3 ± 0.39
Fe	3.44 ± 0.245	8.50 ± 0.177	10.1 ± 0.04	571 ± 4.89
K	1360	894	800	695
Al	2.37 ± 0.264	2.57 ± 0.023	3.47 ± 0.111	6.64 ± 0.499
Si	3.3 ± 1.27	84.8 ± 0.61	81.9 ± 2.23	70.5 ± 0.78

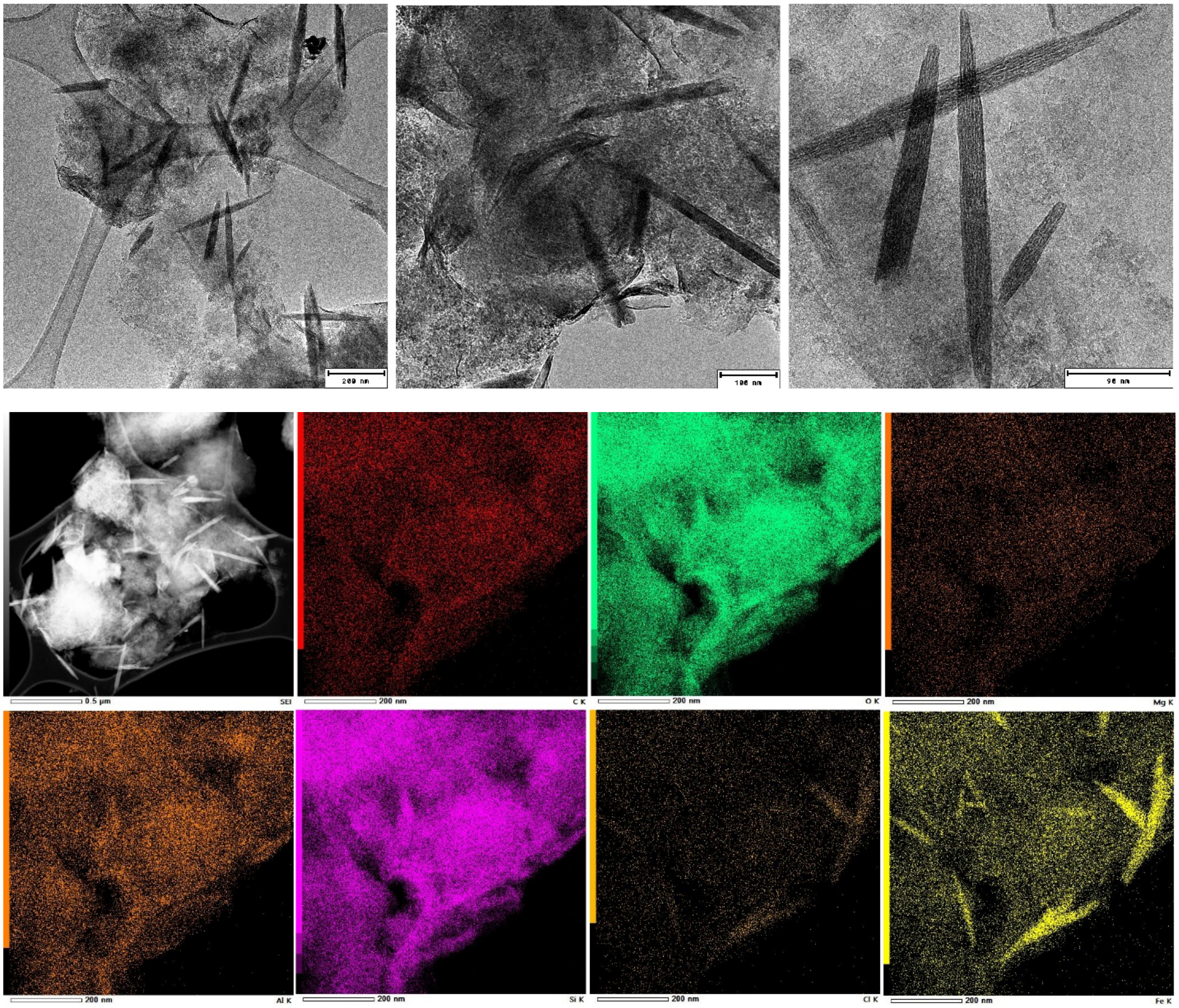

0.5 µm
SEI
200 nm
C K
200 nm
O K
200 nm
Mg K
200 nm
Al K
200 nm
Si K
200 nm
Cl K
200 nm
Fe K

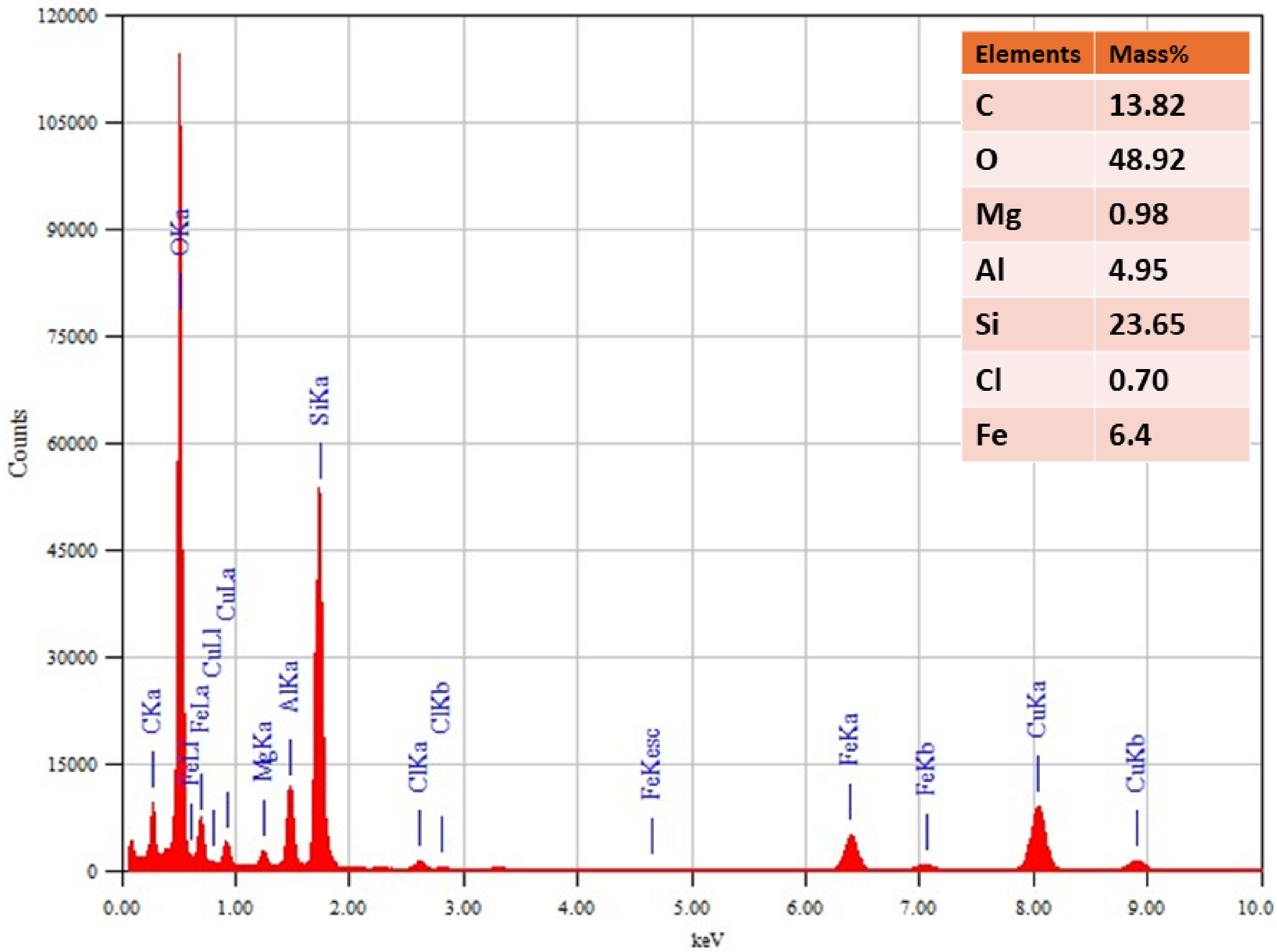

Fe-montmorillonite

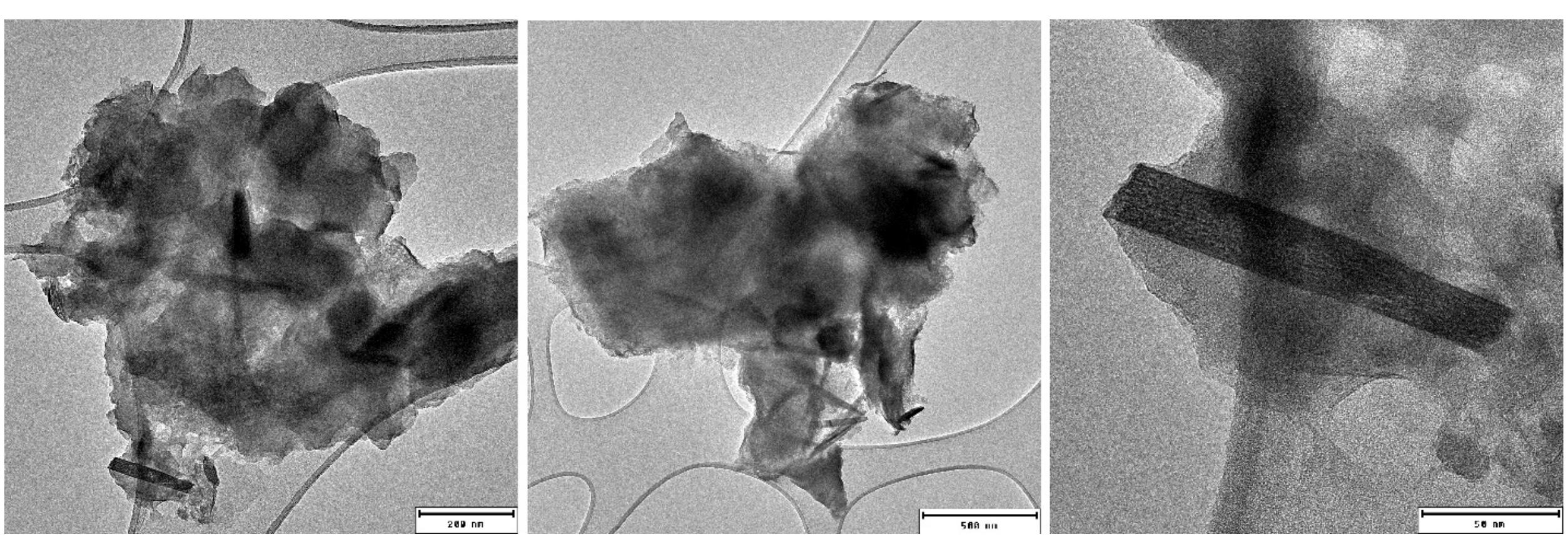

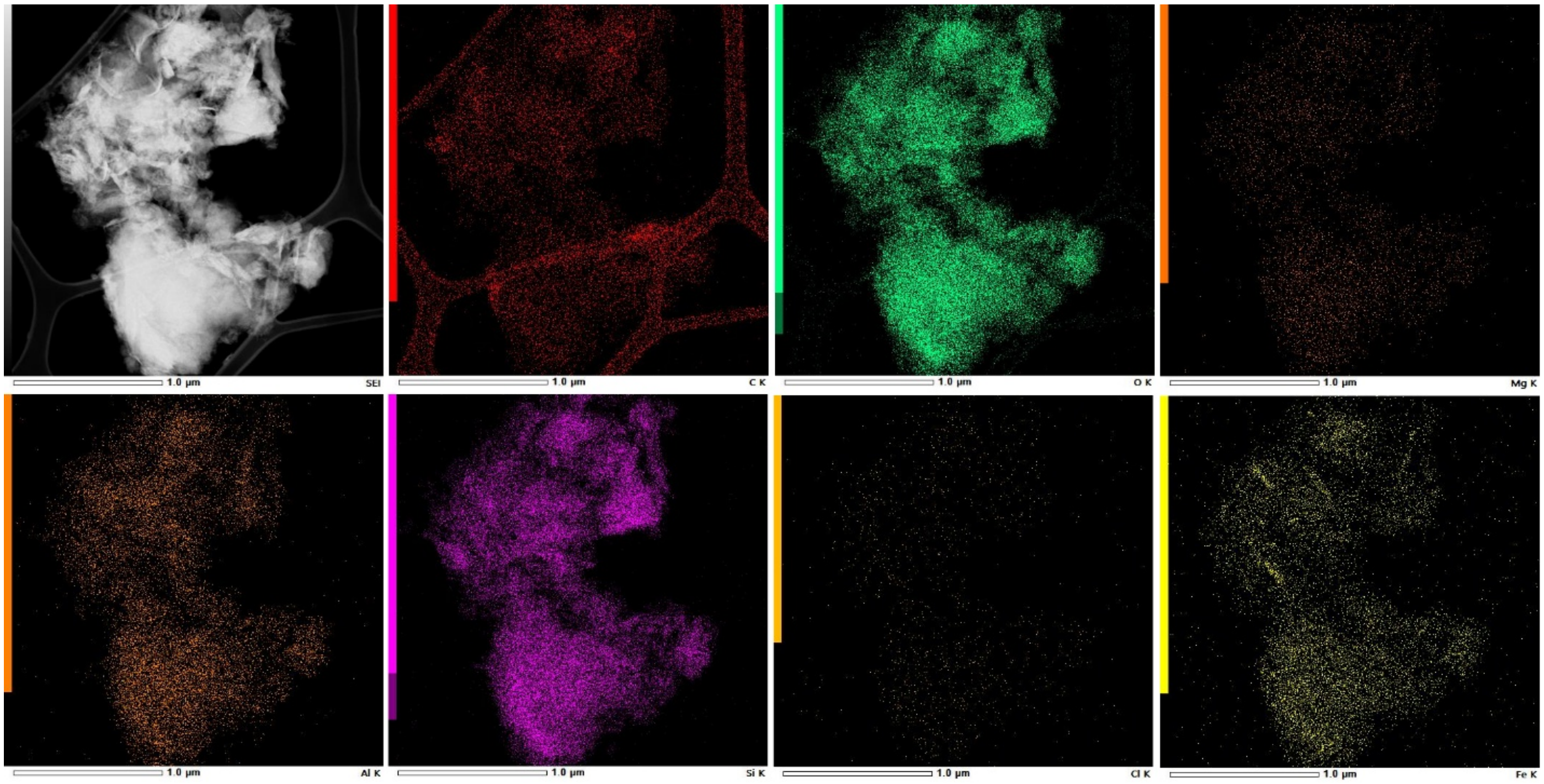

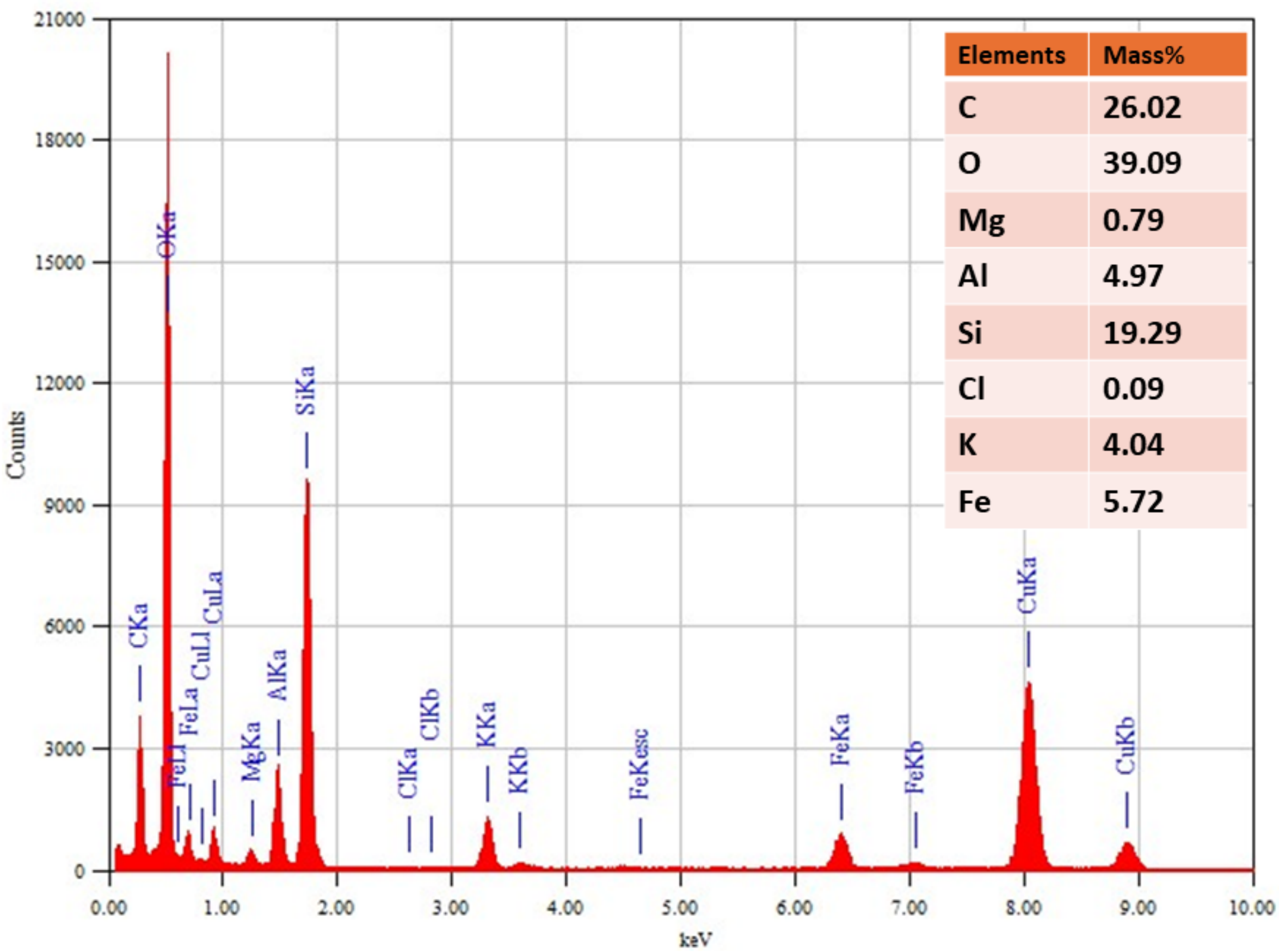

Elements	Mass%
C	26.02
O	39.09
Mg	0.79
Al	4.97
Si	19.29
Cl	0.09
K	4.04
Fe	5.72

Fe-montmorillonite-AHS

Figure S2. STEM images and corresponding EDX elemental maps of Fe-MON before and after AHS adsorption.

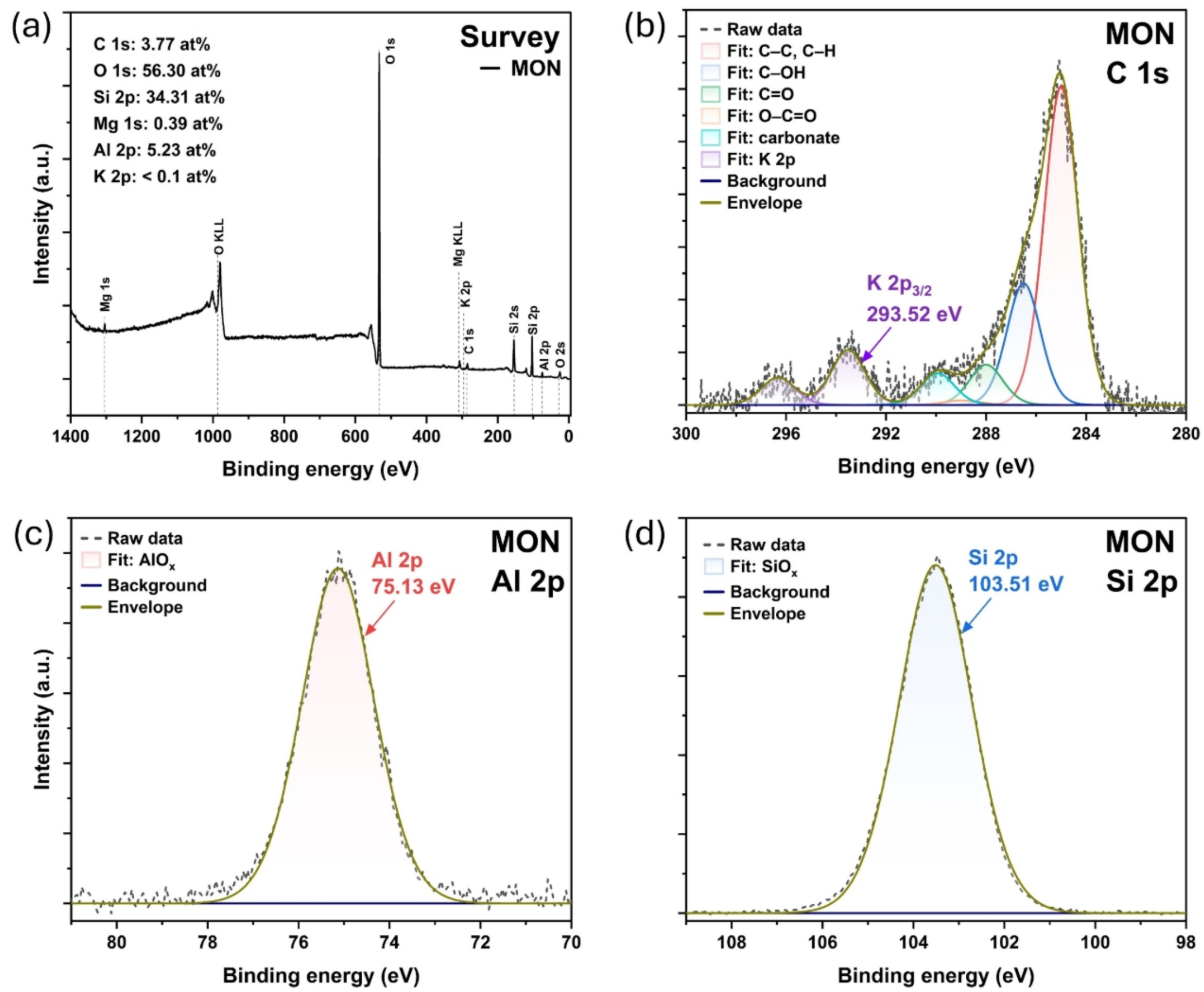

Figure S3. XPS characterization of the reference clay. (a) Survey spectrum showing O, Si, Al, Mg, and C as the principal surface constituents, together with a trace K contribution (<0.1 at.%). High-resolution spectra of (b) C 1s with the overlapping K 2p doublet, (c) Al 2p, and (d) Si 2p. The Al 2p and Si 2p signals were centered at 75.13 and 103.51 eV, respectively, consistent with the aluminosilicate framework of the MON.

The XPS survey spectrum of the reference clay was dominated by O, Si, and Al, as expected for an aluminosilicate material. Carbon accounted for 3.77 at.% and was attributed mainly to adventitious surface carbon. A weak K 2p contribution below 0.1 at.% was also detected, indicating the presence of a minor native or residual potassium-containing component. The Al 2p and Si 2p spectra exhibited single broad components at 75.13 and 103.51 eV, respectively, characteristic of oxidized aluminium and silicon within the clay framework.

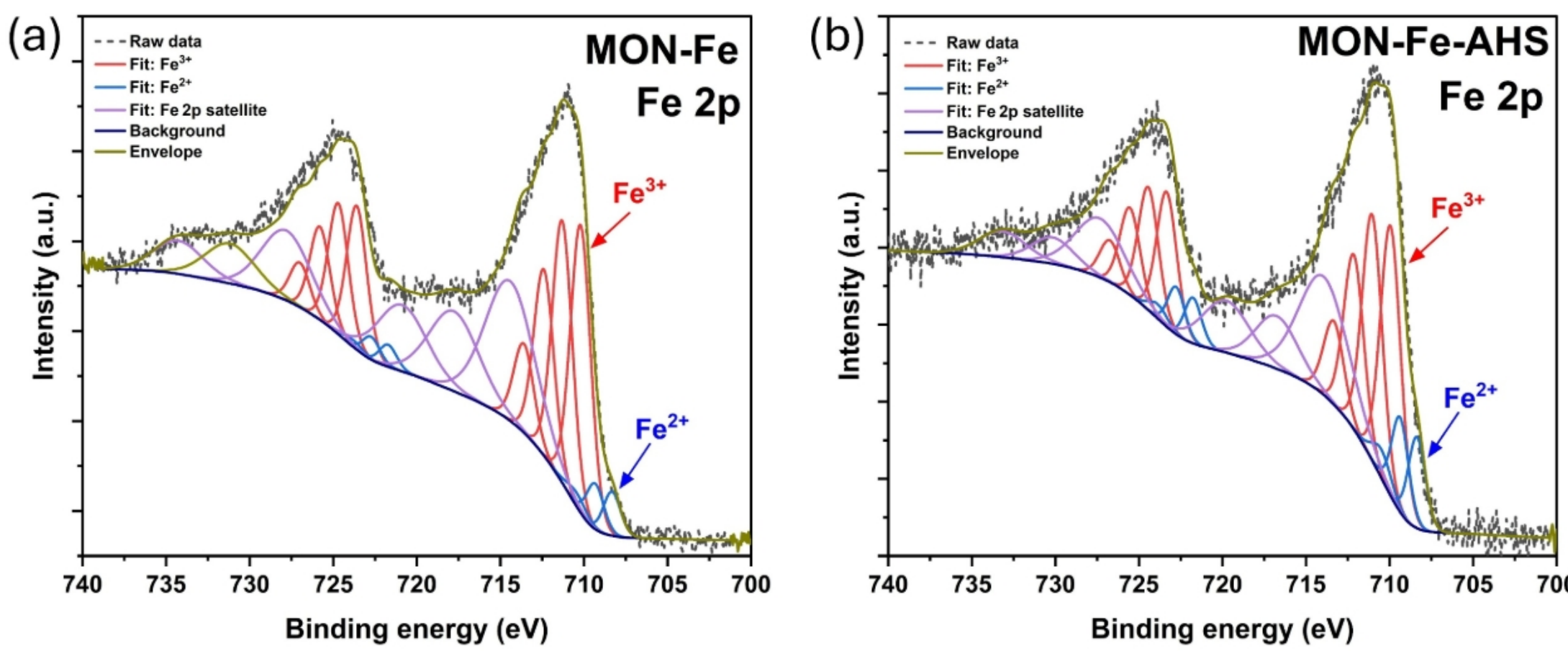

Figure S4. Fe 2p XPS spectra and constrained multiplet fits of (a) MON-Fe and (b) MON-Fe-AHS, showing the Fe^{3+}, Fe^{2+}, and shake-up satellite contributions.

Table S5. Relative Fe^{3+} and Fe^{2+} contributions derived from the fitted Fe 2p main multiplet areas. Shake-up satellite components were excluded from the calculation.

Sample	ΣFe^{3+} area	ΣFe^{2+} area	Fe^{3+} (%)	Fe^{2+} (%)	Fe^{3+}/Fe^{2+} area ratio
MON-Fe-AHS	1488.83	356.20	80.7	19.3	**4.18**
MON-Fe	2720.95	298.72	90.1	9.9	**9.11**

The high-resolution Fe 2p spectra were analysed using a constrained multiplet-fitting procedure based on the approach of Grosvenor et al.[1]. The high-spin Fe^{3+} and Fe^{2+} contributions were represented by

four and three main multiplet components, respectively, with their relative binding-energy separations and peak areas constrained according to the reported reference parameters. The corresponding Fe 2p₁/₂ components were linked to the Fe 2p₃/₂ components using a spin–orbit separation of 13.4 eV and an area ratio of 0.5. All components were fitted using GL(30) line shapes. The fitted FWHM values were 1.20–1.43 eV for the main multiplet components and 2.97–3.50 eV for the broad satellite-related components. The latter were included to account for the additional spectral intensity beyond the principal multiplet structures but were excluded from the calculation of the Fe^{3+}/Fe^{2+} area ratio. The same multiplet constraints were applied to both samples to allow a consistent comparison of their relative Fe oxidation-state contributions. The resulting proportions were therefore treated as model-dependent estimates rather than absolute measurements of Fe speciation.

[1] A. P. Grosvenor, B. A. Kobe, M. C. Biesinger and N. S. McIntyre, Surface and Interface Analysis, 2004, 36, 1564–1574.

Table S6. XPS fitting parameters of the Fe 2p high-resolution spectra.

Peak assignment	Peak ID	MON-Fe BE (eV)	MON-Fe-AHS BE (eV)	Peak constraint	FWHM MON-Fe (eV)	FWHM MON-Fe-AHS (eV)	FWHM (eV) constraint	Area constraint
Fe^{3+} P1 ($2p_{3/2}$)	A	710.2	709.94	A: 709.8–710.4	1.43	1.41	1.2–1.5	A (independent)
Fe^{3+} P2 ($2p_{3/2}$)	B	711.3	711.04	A + 1.1	1.43	1.41	A*1	A * 0.9112
Fe^{3+} P3 ($2p_{3/2}$)	C	712.4	712.14	A + 2.2	1.43	1.41	A*1	A * 0.6476
Fe^{3+} P4 ($2p_{3/2}$)	D	713.6	713.34	A + 3.4	1.43	1.41	A*1	A * 0.3095
Fe^{2+} P1 ($2p_{3/2}$)	E	708.33	708.37	E: 708.2–708.5	1.22	1.2	1.2–1.5	E (independent)
Fe^{2+} P2 ($2p_{3/2}$)	F	709.33	709.37	E + 1.0	1.22	1.2	E*1	E * 1.0385
Fe^{2+} P3 ($2p_{3/2}$)	G	710.43	710.47	E + 2.1	1.22	1.2	E*1	E * 0.3654

Fe^{3+} P1 ($2p_{1/2}$)	H	723.6	723.34	A + 13.4	1.43	1.41	A*1	A * 0.5
Fe^{3+} P2 ($2p_{1/2}$)	I	724.7	724.44	B + 13.4	1.43	1.41	B*1	B * 0.5
Fe^{3+} P3 ($2p_{1/2}$)	J	725.8	725.54	C + 13.4	1.43	1.41	C*1	C * 0.5
Fe^{3+} P4 ($2p_{1/2}$)	K	727	726.74	D + 13.4	1.43	1.41	D*1	D * 0.5
Fe^{2+} P1 ($2p_{1/2}$)	L	721.73	721.77	E + 13.4	1.22	1.2	E*1	E * 0.5
Fe^{2+} P2 (2p₁/₂)	M	722.73	722.77	F + 13.4	1.22	1.2	F*1	F * 0.5
Fe^{2+} P3 (2p₁/₂)	N	723.83	723.87	G + 13.4	1.22	1.2	G*1	G * 0.5
Satellite 1 ($2p_{3/2}$)	O	714.41	713.94	independent	3.5	3.5	2.5–3.5	O (independent)
Satellite 1 ($2p_{1/2}$)	P	727.81	727.34	O + 13.4	3.5	3.5	2.5–3.5	O * 0.5
Satellite 2 ($2p_{3/2}$)	Q	720.88	716.76	independent	3.5	2.97	2.5–3.5	Q (independent)
Satellite 2 ($2p_{1/2}$)	R	734.28	730.16	Q + 13.4	3.5	2.97	2.5–3.5	Q * 0.5
Satellite 3 ($2p_{3/2}$)	S	717.79	719.78	independent	3.5	3.31	2.5–3.5	S (independent)
Satellite 3 ($2p_{1/2}$)	T	731.19	733.18	S + 13.4	3.5	3.31	2.5–3.5	S * 0.5

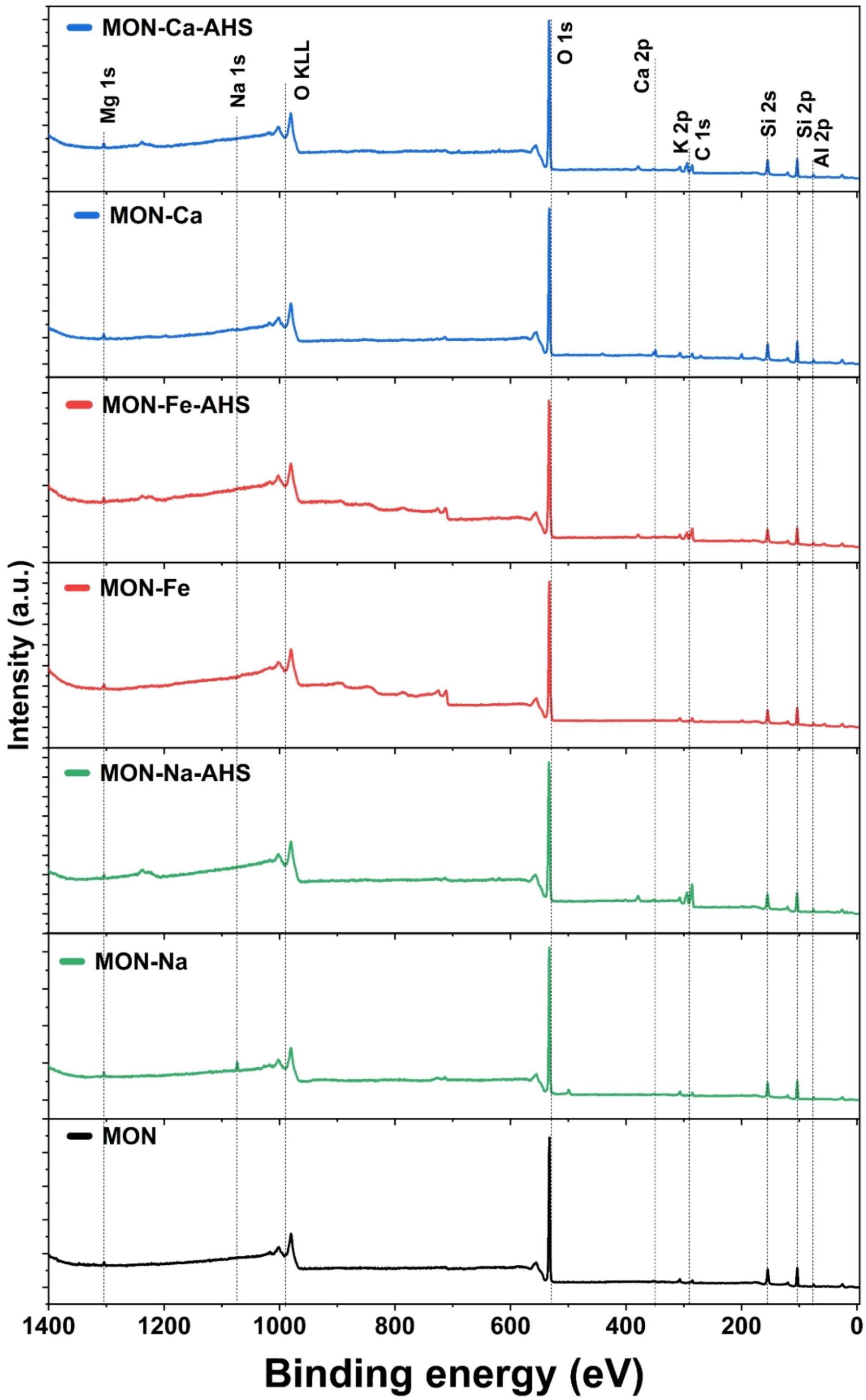

Figure S5. XPS survey spectra of the reference MON, Na-, Ca-, and Fe-modified MON, and the corresponding AHS-treated composites. The principal photoelectron and Auger features are indicated.

Table S7. Quantitative XPS analysis of Na-, Ca-, and Fe-system.

Element	Na system		Ca system		Fe system	
	MON-Na-AHS	MON-Na	MON-Ca-AHS	MON-Ca	MON-Fe-AHS	MON-Fe
**C 1s**	22.94	3.38	9.17	4.86	15.37	4.98
**O 1s**	43.83	57.18	52.65	52.05	51.93	56.26
**Si 2p**	24.37	32.24	27.85	32.74	24.48	29.78
**Al 2p**	4.61	5.49	6.58	5.41	4.32	4.73
**Mg 1s**	0.27	0.47	0.41	0.42	0.27	0.47
**Na 1s**	n.d.	1.06	n.d.	n.d.	n.d.	n.d.
**Ca 2p**	n.d.	n.d.	n.d.	1.71	n.d.	n.d.
**Fe 2p**	n.d.	0.13	n.d.	n.d.	1.41	2.66
**K 2p**	3.43	0.05	3.06	0.24	2.22	0.18
**N 1s**	0.56	n.d.	0.27	n.d.	n.d.	n.d.
**Cl 2p**	n.d.	n.d.	n.d.	2.57	n.d.	0.95

Table S8. Iron extracted from Fe-modified MON (Fe-MON) and pristine MON using hexamminecobalt(III) chloride, acidified KCl, acid ammonium oxalate, and dithionite–citrate-bicarbonate (DCB). Fe concentrations are expressed as mg Fe/g dry solid and reported as mean ± standard deviation.

Extraction method	Element	Fe-MON (mg/g)	MON (mg/g)
Hexamminecobalt(III) chloride	Na	0.054 ± 0.017	0.122 ± 0.003
Hexamminecobalt(III) chloride	Ca	0.167 ± 0.023	1.505 ± 0.035
Hexamminecobalt(III) chloride	Mg	0.084 ± 0.011	0.554 ± 0.011
Hexamminecobalt(III) chloride	K	0.057 ± 0.012	0.131 ± 0.001
Hexamminecobalt(III) chloride	Al	0.480 ± 0.060	0.997 ± 0.018
Hexamminecobalt(III) chloride	Fe	59.3 ± 7.4	1.14 ± 0.06

Acidified KCl	Na	1.148 ± 0.372	2.690 ± 0.594
Acidified KCl	Ca	0.226 ± 0.028	1.430 ± 0.085
Acidified KCl	Mg	0.097 ± 0.011	0.560 ± 0.008
Acidified KCl	Al	0.489 ± 0.046	1.075 ± 0.021
Acidified KCl	Fe	62.8 ± 7.0	0.215 ± 0.006
Acid ammonium oxalate	Na	29.67 ± 11.76	12.00 ± 1.84
Acid ammonium oxalate	Ca	0.112 ± 0.010	0.072 ± 0.006
Acid ammonium oxalate	Mg	0.082 ± 0.005	0.507 ± 0.011
Acid ammonium oxalate	K	24.53 ± 3.25	17.60 ± 0.57
Acid ammonium oxalate	Al	0.455 ± 0.016	1.795 ± 0.050
Acid ammonium oxalate	Fe	54.2 ± 2.5	1.32 ± 0.03
Dithionite–citrate–bicarbonate (DCB)	Ca	0.458 ± 0.077	1.615 ± 0.035
Dithionite–citrate–bicarbonate (DCB)	Mg	0.101 ± 0.006	0.490 ± 0.001
Dithionite–citrate–bicarbonate (DCB)	K	71.10 ± 15.61	58.60 ± 1.98
Dithionite–citrate–bicarbonate (DCB)	Al	0.410 ± 0.028	0.663 ± 0.033
Dithionite–citrate–bicarbonate (DCB)	Fe	58.6 ± 0.8	1.70 ± 0.06

Table S9. Repeated adsorption-desorption cycles of AHS on cation-modified MON.

Material	Cycle	Adsorption capacity (mg C/g)	Desorption capacity (mg C/g)	Residual retained carbon (mg C/g)	Desorption percentage (%)
**Na-MON-AHS**	1	316 ± 15.80	143.15 ± 7.16	172.85 ± 17.35	45.3 ± 3.20%
**Na-MON-AHS**	2	236 ± 11.78	92.04 ± 4.60	143.96 ± 12.67	39.00 ± 2.76%

Na-MON-AHS	3	171 ± 8.55	64.09 ± 3.20	106.91 ± 9.13	37.48 ± 2.65%
Na-MON-AHS	4	109 ± 5.45	40.17 ± 2.01	68.83 ± 5.81	36.85 ± 2.61%
Ca-MON-AHS	1	413 ± 20.63	128.24 ± 6.41	284.76 ± 21.62	31.05 ± 2.20%
Ca-MON-AHS	2	308 ± 15.40	82.24 ± 4.11	225.76 ± 15.94	26.70 ± 1.89%
Ca-MON-AHS	3	262 ± 13.10	58.69 ± 2.93	203.31 ± 13.42	22.40 ± 1.58%
Ca-MON-AHS	4	189 ± 9.45	34.97 ± 1.75	154.03 ± 9.61	18.50 ± 1.31%
Fe-MON-AHS	1	546 ± 27.30	58.37 ± 2.92	487.63 ± 27.46	10.69 ± 0.76%
Fe-MON-AHS	2	391 ± 19.55	35.58 ± 1.78	355.42 ± 19.63	9.10 ± 0.64%
Fe-MON-AHS	3	317 ± 15.84	22.82 ± 1.14	294.18 ± 15.89	7.20 ± 0.51%
Fe-MON-AHS	4	225 ± 11.25	12.83 ± 0.64	212.17 ± 11.27	5.70 ± 0.40%
